# Short-Term Stable Catchment-Level Metabolic Structure in Urban Wastewater Revealed by Untargeted Metabolomics

**DOI:** 10.3390/metabo16100702

**Published:** 2026-09-22

**Authors:** Dandan Li, Yin Wang, Yanfei Shi, Zhen Ma, Yuan Fang, Yan Ma, Bingling Wang, Haiping Duan, Kunzheng Lv

**Affiliations:** 1 Department of Environmental Health, Qingdao Municipal Center for Disease Control and Prevention, Qingdao Institute of Preventive Medicine, No. 175 Shandong Rd., Qingdao 266033, China; ddli87@163.com (D.L.); wangyin0212@126.com (Y.W.); faysy2012@126.com (Y.S.); 2 Qingdao Drainage Operation Service Center, No. 8 Tuandao 3rd Rd., Qingdao 266002, China; mzh2003203386@163.com; 3 Qingdao Eco-Environmental Monitoring Center of Shandong Province, No. 39 Yan’an 1st Rd., Qingdao 266003, China; fangyuan114@163.com; 4 Qingdao Meteorological Bureau (Qingdao Marine Meteorological Bureau), No. 4 Fulongshan Rd., Qingdao 266003, China; qdyanma@163.com; 5 Qingdao Municipal Center for Disease Control and Prevention, Qingdao Institute of Preventive Medicine, No. 175 Shandong Rd., Qingdao 266033, China

**Keywords:** metabolomics, wastewater-based epidemiology, catchment-scale variation, temporal stability, machine learning classification, pharmaceutical biomarkers

## Abstract

**Highlights:**

**What are the main findings?**

- Urban wastewater metabolomes showed short-term stable spatial organization.
- Catchment identity explains 62% of variation with 98% classification accuracy.
- The spatial signal spanned annotated and unannotated features.

**What are the implications of the main findings?**

- Untargeted influent metabolomes provide a stable catchment fingerprint for WBE.
- Spatial structure is not driven solely by obvious anthropogenic tracers.
- Unannotated features carry population-relevant information for marker discovery.

**Abstract:**

**Background:** Urban wastewater integrates chemical signals from human activities and provides a composite biochemical representation of the communities it serves. However, whether wastewater metabolomes exhibit stable spatial organization at the catchment scale remains insufficiently characterized. **Methods:** Untargeted LC–HRMS was applied to 56 influent wastewater samples collected from seven urban catchments over eight consecutive days to evaluate spatial structuring of the community metabolome. **Results:** A total of 3248 reproducible molecular features were detected. Permutational multivariate analysis of variance indicated that catchment identity explained 62% of total metabolomic variation (R^2^ = 0.62, *p* < 0.001), while multivariate dispersion did not differ significantly among sites (*p* = 0.082). Within-catchment compositional distances were significantly lower than between-catchment distances (*p* < 0.001), and centroid separation exceeded internal dispersion (ratio = 2.55), indicating short-term stable, site-specific configurations in high-dimensional chemical space. Random Forest classification achieved 98% accuracy under strict spatial cross-validation and 98.2% under leave-one-day-out validation (*p* < 0.001). Classification performance remained high using annotated-only (98.2%), unannotated-only (98.2%), or pharmaceutical-excluded datasets (96.5%), demonstrating that spatial identity was encoded in a distributed manner across the metabolome rather than driven by a limited subset of compounds. **Conclusions:** These findings provide quantitative evidence that urban wastewater metabolomes exhibit short-term stable, reproducible catchment-level structure and establish a methodological foundation for system-level characterization of urban populations using untargeted metabolomics.

## 1. Introduction

Urban wastewater represents a chemically integrated matrix of human activities. Domestic discharge, commercial inputs, dietary metabolites, pharmaceuticals, personal care products, and endogenous biochemicals collectively converge into sewer networks, forming a pooled molecular signature of community-level exposure [1,2,3]. Because each wastewater treatment plant (WWTP) aggregates inputs from large residential populations, influent wastewater constitutes a composite biochemical profile representative of the communities it serves. This integrative property has positioned wastewater as a valuable resource for epidemiological surveillance, chemical exposure assessment, and public health monitoring [1,4,5]. Applications such as wastewater-based epidemiology have demonstrated the feasibility of quantifying population-scale consumption of illicit drugs, pharmaceuticals, and other target compounds [6,7,8,9].

Despite these advances, most wastewater monitoring approaches remain compound-centric, focusing on predefined chemical classes or individual biomarkers [10]. While such targeted analyses provide quantitative estimates of specific exposures, they capture only a small fraction of the total chemical complexity present in wastewater. Untargeted high-resolution mass spectrometry has revealed that wastewater metabolomes comprise thousands of molecular features spanning endogenous metabolites, anthropogenic chemicals, transformation products, and yet-unannotated compounds [11,12]. However, the large-scale organization of this high-dimensional chemical space remains poorly characterized [13,14].

An open question is whether urban wastewater metabolomes exhibit stable spatial organization at the catchment scale, where each catchment corresponds to the population served by an individual WWTP. Previous studies have reported spatial variability in wastewater chemical composition across cities or treatment plants [15,16,17], yet it remains unclear whether such variability reflects reproducible structural differentiation or transient fluctuations driven by hydrological and operational factors [18]. In contrast to these earlier reports, which were largely based on targeted compound panels and single or infrequent sampling, the present study applies untargeted LC–HRMS with repeated daily sampling at the catchment scale. This design allows explicit evaluation of whether catchment-level spatial differentiation is temporally reproducible and independent of variance heterogeneity. If wastewater truly integrates community-specific metabolic and behavioral patterns, one would expect catchments to occupy distinct and reproducible regions in high-dimensional chemical space. Alternatively, the metabolome may be dominated by stochastic variability, hydrological fluctuations, or transient exogenous inputs, limiting spatial reproducibility [19,20]. Distinguishing between these possibilities is essential for evaluating whether wastewater metabolomics can support robust community-level characterization beyond single-compound analysis.

Spatial differentiation in complex environmental datasets can arise through two fundamentally different mechanisms: shifts in multivariate location (systematic compositional differences) or differences in dispersion (variance structure). In ecological and environmental metabolomics studies, apparent group separation is sometimes driven by unequal variability rather than true compositional structuring [21,22]. Therefore, rigorous evaluation of both multivariate location and dispersion is necessary to establish meaningful catchment-level organization.

Furthermore, even when spatial differences are detectable, it remains unclear whether such differentiation is driven primarily by a limited set of dominant anthropogenic chemicals (e.g., pharmaceuticals) or encoded more broadly across the metabolome [23,24]. If classification performance relies heavily on a small subset of annotated compounds, spatial discrimination may reflect differential usage patterns of specific products. In contrast, distributed contributions across both annotated and unannotated molecular features would suggest that catchment identity emerges from systemic metabolic configurations rather than isolated chemical markers.

High-dimensional statistical learning provides a complementary framework for evaluating spatial structure [25]. Cross-validated classification models can quantify group separability among communities while minimizing overfitting [26], and permutation-based validation is widely recommended to assess model significance in metabolomics datasets [27]. Robust validation strategies, such as grouped or leave-one-time-point-out cross-validation, are particularly important when repeated measures or temporal autocorrelation are present [28].

We hypothesized that influent wastewater metabolomes from different urban catchments occupy distinct and reproducible regions of high-dimensional chemical space, and that this spatial separation (i) remains stable over the eight consecutive sampling days, (ii) reflects shifts in multivariate location rather than differences in dispersion, and (iii) is encoded distributively across the metabolome rather than driven by a limited set of dominant compounds. In this study, we investigated whether urban wastewater metabolomes exhibit stable, catchment-level spatial organization using untargeted liquid chromatography–high-resolution mass spectrometry (LC–HRMS). Wastewater samples were collected from seven urban WWTP catchments over eight consecutive days to capture short-term temporal dynamics while enabling evaluation of spatial reproducibility. We first characterized overall compositional variation and quantified the contribution of catchment identity using permutational multivariate analysis of variance (PERMANOVA) [27], alongside tests for homogeneity of dispersion [29], thereby distinguishing centroid separation from dispersion effects. We then assessed the stability of site-specific configurations through centroid analysis and intra- versus inter-catchment distance comparisons. Together, these analyses provide quantitative evidence that catchment-level metabolic structure is stable over the eight-day sampling window—to our knowledge, the first such demonstration in urban wastewater—and is robust to analytical and biological perturbations.

To further evaluate high-dimensional separability, we implemented Random Forest classification under strict cross-validation frameworks, including GroupKFold and leave-one-day-out validation [30,31]. We examined the distribution of feature importance to determine whether spatial discrimination was concentrated in a small subset of compounds or broadly distributed across the metabolome. Finally, we evaluated the relative contributions of annotated and unannotated features and tested robustness after removal of pharmaceutical compounds to determine whether catchment identity depended predominantly on a limited set of exogenous chemical inputs.

## 2. Materials and Methods

### 2.1. Study Design and Sample Collection

Influent wastewater samples were collected from seven municipal wastewater treatment plants (WWTPs) using 24-h flow-proportional composite sampling. Eight consecutive daily samples were obtained from each plant (*n* = 56 total). Basic characteristics of the seven catchments, including served population, daily influent flow (during the sampling period), and predominant wastewater composition, are summarized in Table S1. Populations served ranged from ~45,000 to ~1.38 million, and daily influent flows ranged from ~4600 to ~293,000 m^3^/d. Most catchments were predominantly residential, whereas the LC catchment additionally received industrial wastewater. Samples were transported on ice and stored at −80 °C until analysis.

### 2.2. Sample Preparation

Frozen samples were thawed at 4 °C and homogenized. A 5 mL aliquot of each sample was evaporated to dryness under vacuum. Residues were extracted with 1 mL pre-cooled methanol/water (4:1, *v*/*v*), sonicated for 20 min in an ice bath, and incubated at −20 °C for 1 h to facilitate protein precipitation. After centrifugation (16,000× *g*, 4 °C, 20 min), supernatants were collected and evaporated to dryness. Extracts were reconstituted in 400 µL methanol/water (1:1, *v*/*v*), centrifuged (20,000× *g*, 4 °C, 15 min), and transferred to autosampler vials for LC–MS analysis. Biological samples were injected in a randomized order, with five injections of a pooled quality control (QC) sample (prepared by equal-volume mixing of all biological samples) interspersed throughout the analytical sequence to monitor instrument stability and reproducibility. As no separate blank injections were performed, signal reliability was controlled via the pooled QC samples and a strict feature-level reproducibility filter. Across the five QC injections, feature intensities showed a median RSD of 8.90% (maximum 30.00%), and all 3248 features retained for analysis passed the 30% RSD threshold. PCA of the full sample set showed tight clustering of the QC samples (PC1 = 16.69%, PC2 = 10.45%; QC distance to centroid = 1.22 ± 0.74, Figure S1).

### 2.3. LC–HRMS Analysis

Untargeted metabolomic profiling was performed using a Shimadzu LC-30 UHPLC system (Shimadzu Corporation, Kyoto, Japan) coupled to a Q Exactive Plus Orbitrap mass spectrometer (Thermo Scientific, Waltham, MA, USA) equipped with a heated electrospray ionization source.

Chromatographic separation was achieved on an ACQUITY UPLC® HSS T3 column (2.1 × 100 mm, 1.8 µm; Waters Corporation, Milford, MA, USA) maintained at 40 °C with a flow rate of 0.3 mL min^−1^. Mobile phases consisted of water with 0.1% formic acid (A) and acetonitrile with 0.1% formic acid (B). A linear gradient from 2% to 100% B was applied over 13 min, followed by re-equilibration (total run time 15 min).

Mass spectra were acquired in positive and negative ionization modes over *m*/*z* 75–1050 at 70,000 resolution (*m*/*z* 200). Data-dependent MS/MS fragmentation was performed on the top 10 most intense ions using stepped collision energies. 

Principal component analysis (PCA) of QC samples was used to verify analytical stability and absence of systematic drift.

All 56 samples were extracted and processed in a single batch. The injection sequence was randomized with respect to catchment and sampling day to prevent confounding of spatial structure with analytical order, and pooled QC samples were injected at regular intervals throughout the sequence to monitor instrument stability and drift.

### 2.4. Data Processing and Metabolite Annotation

Raw data files were processed using MS-DIAL (v4.9) for peak detection, retention time alignment, and feature quantification. Features were aligned across all samples based on accurate mass and retention time.

Putative annotations were assigned by matching accurate mass (<20 ppm) and MS/MS spectra against HMDB, MassBank, GNPS, and an in-house spectral library. Annotation confidence was graded on a four-tier scale: Level 1 (accurate mass + MS/MS + retention time; highest confidence), Level 2 (accurate mass + MS/MS), Level 3 (accurate mass + retention time), and Level 4 (accurate mass only). Only the two highest tiers (Levels 1 and 2), i.e., annotations supported by MS/MS spectral evidence, were retained; no Level 3 or Level 4 features passed the quality filter. Among the 3248 retained features, 2332 (71.8%) were assigned Level 1 and 916 (28.2%) Level 2. In MSI terms, all retained annotations correspond to putatively identified compounds (MSI Level 2); no authentic-standard–confirmed (MSI Level 1) assignments are claimed. Features that additionally received a confident chemical-class (Superclass) assignment (*n* = 2436; 75.0%) are hereafter referred to as “annotated,” whereas the remaining putatively identified features lacking a definitive chemical-class assignment (*n* = 812; 25.0%) are referred to as “unannotated.”

Features with >50% missing values within any catchment were excluded. Features exhibiting relative standard deviation (RSD) > 30% across the five QC injections were removed to ensure analytical robustness. Applying this filter retained 3248 features with a median QC RSD of 8.90%. Signal intensities were normalized to total ion current, log10-transformed, and standardized (z-score) prior to statistical analysis. Data from positive and negative ionization modes were merged after normalization.

### 2.5. Statistical Analysis

Bray–Curtis dissimilarities were calculated from z-score-standardized feature intensities (after total-ion-current normalization and log10 transformation). Although z-score standardization produces negative values and Bray–Curtis dissimilarities computed on such data no longer retain the 0–1 range of the original abundance-based definition, all downstream inferences (PERMANOVA, PERMDISP, Wilcoxon rank-sum tests, and permutation tests) are based on the rank ordering of the dissimilarity matrix and are therefore insensitive to this scale shift. This standardization ensures equal weighting across features of highly variable intensity, preventing high-abundance features from dominating the dissimilarity estimates. Global metabolomic structure was explored using principal coordinate analysis (PCoA). Catchment effects were assessed using PERMANOVA (9999 permutations). Homogeneity of multivariate dispersion was evaluated using PERMDISP (9999 permutations), computed from the distances of samples to their own catchment centroid.

Within- and between-catchment dissimilarities were compared using Wilcoxon rank-sum tests. Two complementary distance-based ratios were computed: (i) the pairwise distance ratio, defined as the mean between-catchment dissimilarity divided by the mean within-catchment dissimilarity and computed from all pairwise sample distances, with statistical significance assessed by permutation testing (9999 label randomizations); and (ii) the centroid separation ratio, defined as the mean inter-centroid distance divided by the mean within-catchment dispersion, where catchment centroids were computed in Bray–Curtis space and within-catchment dispersion is the mean distance of samples to their own catchment centroid. 

Supervised classification was performed using Random Forest (1000 trees) and Support Vector Machine models with catchment identity as the response variable. Two group-structured cross-validation schemes were applied to control for the non-independence of repeated samples from the same day or catchment. In leave-one-day-out (LODO) validation, all samples from one of the eight sampling days were held out as the test set and the model was trained on the remaining days, testing whether spatial discrimination is preserved across time. In leave-one-site-out (LOSO) validation, all samples from one catchment were held out, and the model was trained on the remaining catchments, testing whether the learned patterns generalize to an unseen catchment. Statistical significance was assessed by permutation testing (1000 iterations) under the LODO scheme. Performance was measured as overall accuracy and macro-averaged F1 score.

Random Forest feature importance was quantified using mean decrease in impurity. Stability of top-ranked features was evaluated across cross-validation folds, and features appearing in ≥70% of folds were considered stable contributors. Chemical category enrichment among top-ranked features was assessed using Fisher’s exact tests with Benjamini–Hochberg false discovery rate correction.

Variance partitioning was conducted using linear mixed-effects models to estimate contributions of catchment and sampling day. Sensitivity analyses included removal of annotated pharmaceuticals, removal of industrial/consumer-associated features, balanced subsampling of annotated and unannotated features, bootstrap resampling, and exclusion of outlier sampling days. 

All analyses were performed in Python (v3.11) using pandas, numpy, scipy, and scikit-learn. Statistical significance was defined at α = 0.05 unless otherwise specified.

## 3. Results

### 3.1. Spatial Organization of the Urban Wastewater Metabolome

Untargeted LC–HRMS analysis of 56 wastewater samples collected from seven urban catchments over eight consecutive days yielded 3248 reproducibly detected molecular features after quality filtering. Detected features included annotated pharmaceuticals, personal care products, endogenous metabolites, and a substantial proportion of unannotated compounds, reflecting broad chemical diversity.

Principal coordinate analysis (PCoA) based on Bray–Curtis dissimilarity revealed clear clustering of samples according to catchment (Figure 1A). PERMANOVA confirmed that catchment identity explained a significant fraction of compositional variance (R^2^ = 0.62, *p* < 0.001, Table S2). Analytical stability was confirmed by pooled QC samples (*n* = 5) injected throughout the sequence, which showed a median per-feature relative standard deviation (RSD) of 2.5% (mean 3.7%); among the 3248 retained features, the median QC RSD was 8.9% (95th percentile 24.5%), well below the 30% filtering threshold. PCA of QC samples confirmed tight clustering with no systematic drift across the randomized injection sequence. Multivariate dispersion did not differ significantly among catchments (PERMDISP, *p* = 0.082; Figure 1B, Table S3), indicating that observed group separation was attributable primarily to shifts in multivariate centroid position rather than heterogeneity in dispersion. Variance partitioning across the 3248 features showed that catchment identity explained 37.0% ± 23.0% of per-feature variance on average, sampling day explained 8.0% ± 12.2%, and residual variation accounted for 54.9% ± 22.1% (catchment-to-day variance ratio = 4.6; catchment exceeded day in 83.5% of features), quantitatively confirming that spatial organization dominates day-to-day temporal variation.

### 3.2. Temporal Stability of Catchment-Specific Signatures

To assess temporal consistency, Bray–Curtis dissimilarities were compared within and between catchments across eight sampling days. Within-catchment distances (mean = 0.651) were significantly lower than between-catchment distances (mean = 1.072; Wilcoxon test, *p* < 0.001, Cohen’s d = 2.19; Figure 2A), yielding a distance ratio of 1.65. In multivariate space, daily samples from the same catchment clustered tightly around site-specific centroids (mean sample-to-centroid dispersion = 0.47), while centroids of different catchments remained clearly separated (mean inter-centroid distance = 1.20; Figure 2B). The ratio of mean inter-centroid distance to mean within-catchment dispersion was 2.55, demonstrating that spatial separation substantially exceeded internal temporal variability.

### 3.3. High-Dimensional Separability of Catchments

To evaluate the discriminative power of metabolic fingerprints, we applied random forest classification using leave-one-day-out cross-validation (Table S4). The model achieved a mean accuracy of 98.2% ± 4.7% across eight validation folds (Figure 3A), substantially exceeding the random baseline accuracy of 14.3% expected for seven equally represented classes. High accuracy was consistently observed across held-out days, indicating that spatial discrimination was robust to short-term temporal variability. Across the eight leave-one-day-out folds, 188 of the 324 top-10% ranked features, 42 of the top 100, and 6 of the top 20 features were consistently ranked (present in ≥70% of folds; Table S5), indicating a stable set of catchment-discriminative molecular features. In contrast, when an entire catchment was held out (LOSO), classification accuracy was 0% for all seven held-out catchments (Table S6). This indicates that the discriminative patterns are catchment-specific composite signatures that do not generalize to an unseen catchment, consistent with the site-specific metabolic organization documented above. 

Feature importance was unevenly distributed across the metabolome rather than confined to a small subset of predictors. The Lorenz curve deviated markedly from the line of perfect equality (Gini coefficient = 0.75; Figure 3B, Table S7), indicating moderate concentration of predictive importance. The top 10% and 20% of ranked features accounted for 53.9% and 76.5% of total model importance, respectively, suggesting that classification performance emerged from a partially concentrated yet intrinsically high-dimensional feature structure.

Permutation testing (1000 iterations) showed that no permuted dataset achieved an accuracy equal to or greater than the observed value (0.98), corresponding to *p* ≤ 0.001 (Figure 3C). Together, these results demonstrate that catchment-specific metabolomic signatures are strongly separable in high-dimensional feature space and that predictive performance cannot be attributed to random variation. Bootstrap resampling (200 iterations) yielded an out-of-bag accuracy of 92.6% (95% CI 74.9–100%), and classification reached 100% (49/49) after excluding the single outlier sampling day (day 6) on which one sample was misclassified, confirming the robustness of the spatial signal.

### 3.4. Independent Contributions of Annotated and Unannotated Metabolites

To evaluate the relative contributions of chemically annotated and unannotated features, classification models were trained separately using annotated metabolites (*n* = 2436), unannotated features (*n* = 812), and the combined dataset (*n* = 3248). Remarkably, all three models achieved identical mean classification accuracy (98.2%; Figure 4A). The consensus confusion matrix further demonstrated highly consistent prediction patterns across feature subsets, with only a single recurrent misclassification (MD classified as LC; Figure 4B). These results indicate that both annotated and unannotated fractions independently contain sufficient discriminatory information, supporting the presence of a distributed spatial signal across the metabolome rather than reliance on a limited subset of chemically characterized metabolites.

### 3.5. Spatial Structure Independent of Pharmaceutical Signals

To determine whether spatial discrimination was driven primarily by differential pharmaceutical usage, all annotated pharmaceutical compounds were removed, and classification was repeated. Model accuracy remained high at 96.5% (Figure 5), indicating that catchment separability was not solely attributable to pharmaceutical signatures. The small reduction in accuracy (1.7%) compared to the full dataset (98.2%) was not statistically significant (Wilcoxon signed-rank test, *p* = 1.000), suggesting that pharmaceutical compounds did not contribute disproportionately to spatial discrimination. To evaluate whether other anthropogenic chemical classes acted as competing drivers, the classification was repeated after removal of all 169 features assigned to the industrial consumer category and, separately, using only the 2695 features that could not be assigned to any source category (i.e., features of unknown source provenance). Accuracy remained high in both cases (96.4% and 98.2%, respectively), demonstrating that the discriminating signal was not dependent on industrial or consumer-product inputs and was encoded in a distributed manner across the untargeted metabolome.

### 3.6. Centroid-Driven Spatial Differentiation

PERMDISP indicated no significant differences in multivariate dispersion among catchments (F = 1.88, *p* = 0.082; Figure 1B), suggesting comparable within-catchment variability. Between-catchment distances were significantly greater than within-catchment distances (Wilcoxon rank-sum test, *p* < 0.001), indicating clear spatial separation despite similar dispersion patterns. The mean inter-centroid distance (1.20) substantially exceeded the mean within-catchment dispersion (0.47), resulting in a centroid separation ratio of 2.55. This centroid-based separation was further supported by high cross-validated classification accuracy (98.2% for the full dataset; 96.5% after removal of pharmaceuticals), which remained stable across temporal subsets.

## 4. Discussion

This study demonstrates that influent wastewater metabolomes from seven municipal WWTP catchments exhibit reproducible spatial organization at the community level. Across distance-based analyses and supervised classification, between-catchment differentiation consistently exceeded within-catchment temporal variability, indicating that wastewater metabolomic composition contains stable structural signatures associated with the populations served by each WWTP. Given that wastewater integrates collective human activity at the population scale [1,32,33], these findings extend prior compound-specific applications of wastewater-based epidemiology [34,35,36,37] by demonstrating organization at the level of the untargeted metabolome.

Variance partitioning showed that catchment identity explained, on average, 4.6 times more per-feature variance than sampling day (37.0% vs. 8.0%), supporting the existence of temporally stable multivariate centroids. Importantly, tests for homogeneity of dispersion confirmed that spatial separation was not solely attributable to unequal variability among groups. Apparent clustering in high-dimensional environmental datasets can arise from dispersion artifacts rather than true compositional shifts [38,39]; thus, explicitly distinguishing multivariate location from dispersion effects is critical. Together, these results indicate that communities occupy distinct regions in high-dimensional chemical space rather than differing only in variance structure.

Because each WWTP aggregates contributions from large residential populations [40,41,42], the observed differentiation is consistent with community-scale integration, although it cannot be attributed solely to population composition. Wastewater composition may also be shaped by catchment-specific infrastructure (e.g., sewer network characteristics, treatment plant configuration), industrial and commercial inputs, and hydrological conditions, which were not independently characterized here. Nevertheless, aggregation across thousands of contributors likely dampens short-term stochastic fluctuations while preserving persistent structural characteristics of the served catchment. In this sense, the resulting metabolomic configuration can be interpreted as an emergent, catchment-level chemical signature—a composite profile that integrates the daily exposures of the served population and remains stable across sampling days—consistent with the integrative framework underlying wastewater-based monitoring [5,43].

Supervised classification under strict GroupKFold and leave-one-day-out validation demonstrated robust separability without temporal leakage. Rigorous cross-validation is essential in high-dimensional datasets to prevent overfitting and inflated performance estimates, particularly in metabolomics applications [44,45,46]. Although the present dataset is high-dimensional relative to its sample size (3248 features vs. 56 samples)—a setting in which overfitting is a legitimate concern—all reported accuracies derive from strictly out-of-sample validation: under leave-one-day-out and day-grouped GroupKFold schemes, an entire sampling day was withheld in each fold, and the model was retrained from scratch, such that no feature selection, tuning, or permutation step ever involved the held-out samples. Permutation testing (1000 iterations) under this identical scheme yielded null accuracies far below the observed performance (mean ≈ 0.11, near the chance-level baseline of 0.143 for seven classes; *p* ≤ 0.001), and the discriminating signal was distributed across the metabolome rather than concentrated in a few features: 49% of the 3248 features carried non-zero importance, annotated-only and unannotated-only models each reproduced 98.2% accuracy, and accuracy remained high (96.5%) after removal of 294 annotated pharmaceuticals. Model complexity was additionally constrained by limiting tree depth, and the convergence of these independent lines of evidence argues against overfitting or data leakage as explanations for the observed accuracy. Given the relatively small dataset (*n* = 56 samples), the high classification accuracies should be interpreted as evidence of strong multivariate separability among the seven sampled catchments rather than as predictive generalization to unsampled catchments or time periods. This distributed encoding suggests that catchment identity emerges from systemic organization of the wastewater metabolome rather than isolated chemical markers, aligning with recent perspectives on non-target chemical fingerprinting in environmental matrices [23,47,48].

Importantly, removal of pharmaceutical features did not eliminate spatial separability, indicating that structure was not solely driven by obvious anthropogenic tracers. While pharmaceuticals are commonly used as targeted indicators in wastewater studies [49,50,51], the persistence of differentiation after their exclusion suggests that catchment-level structure is embedded broadly across the untargeted chemical profile captured by LC–HRMS [52,53,54].

Beyond their aggregate statistical contribution, the annotated compounds most vital to catchment separation can also be interpreted in terms of their release, biotransformation, occurrence, and fate within the urban drainage system, which adds chemical and toxicological meaning to the spatial structure documented above. The top-ranked discriminative features encompassed human biotransformation products of pharmaceuticals—including the major active metabolite of losartan (E-3174, annotated as losartan carboxylic acid), 4′-hydroxydiclofenac (the major oxidative metabolite of diclofenac), clopidogrel carboxylic acid, and 4-acetamidoantipyrine (a metamizole metabolite)—together with unmetabolized parent drugs (e.g., atenolol, telmisartan, pirenzepine), host–microbiome co-metabolites and endogenous conjugates (e.g., phenylacetylglutamine), and industrial and consumer chemicals such as the persistent artificial sweetener sucralose, the plasticizer metabolite monoethyl phthalate, the flame retardant tris(2-chloroisopropyl)phosphate, and cyclic siloxanes. Because all samples were collected at wastewater treatment plant inlets, each feature integrates chemical release across the served catchment, and its contribution to catchment separation depends on the continuity and magnitude of emission as well as on its stability during sewer conveyance: continuously discharged and transformation-resistant markers such as sucralose are expected to yield stable catchment fingerprints, whereas more labile compounds are attenuated or converted by (bio)transformation—including microbial metabolism in sewer biofilms—before reaching the plant [36]. The co-detection of parent–metabolite pairs such as losartan/E-3174 and diclofenac/4′-hydroxydiclofenac further indicates that parent compounds and their metabolic products co-occur in influent wastewater, and their relative abundance jointly encodes human metabolism and in-sewer stability, information directly relevant to marker selection in future quantitative wastewater-based epidemiology applications [36,49,50,51]. Considered together, the annotated compounds driving catchment separation trace a continuum from chemical release—human, microbial, or industrial—to (bio)transformation and fate during transport in the sewer network, and this source-to-fate perspective should inform the interpretation of discriminative features in wastewater metabolomic fingerprints [49,50,51].

Several limitations warrant consideration. The modest sample size (56 samples from seven catchments over eight days) warrants caution when interpreting the very high classification accuracies, which primarily reflect within-dataset group separability rather than generalizable predictive performance. The study was conducted over a defined temporal window, while the present data support short-term (eight-day) stability, as demonstrated by leave-one-day-out cross-validation and variance partitioning; longer-term monitoring is needed to evaluate seasonal persistence and stability across hydrological conditions [55,56,57]. Additionally, untargeted LC–HRMS captures only a fraction of the accessible chemical space [14,58,59], and structural annotation remains incomplete. Consequently, mechanistic interpretation of discriminatory features is limited. Direct data on daily flow rates, served population sizes, and sewer infrastructure characteristics (e.g., pipe age, hydraulic retention time, in-sewer transformation) were not available in this study, and their potential contribution to the observed differentiation therefore cannot be definitively excluded. Of note, all samples were collected as 24 h flow-proportional composites, which largely normalizes within-sample flow and dilution variability, and per-feature z-score standardization removes multiplicative between-sample scaling differences; nevertheless, infrastructure-mediated effects remain a recognized competing explanation. Future catchment-scale studies should collect such metadata to allow formal testing of these factors. However, the primary objective of this study was not compound-level attribution but evaluation of high-dimensional spatial organization.

## 5. Conclusions

By rigorously distinguishing centroid separation from dispersion artifacts and validating classification under strict cross-validation frameworks, this work provides quantitative evidence that urban wastewater metabolomes exhibit reproducible catchment-level structure that remained stable across the eight-day sampling window. Establishing the existence and reproducibility of such structure represents a necessary first step toward advancing wastewater metabolomics from compound-specific surveillance to system-level characterization of urban communities. Broader conclusions regarding community-level characterization, however, must await validation across longer time periods and additional cities/catchments.

## Figures and Tables

**Figure 1:**
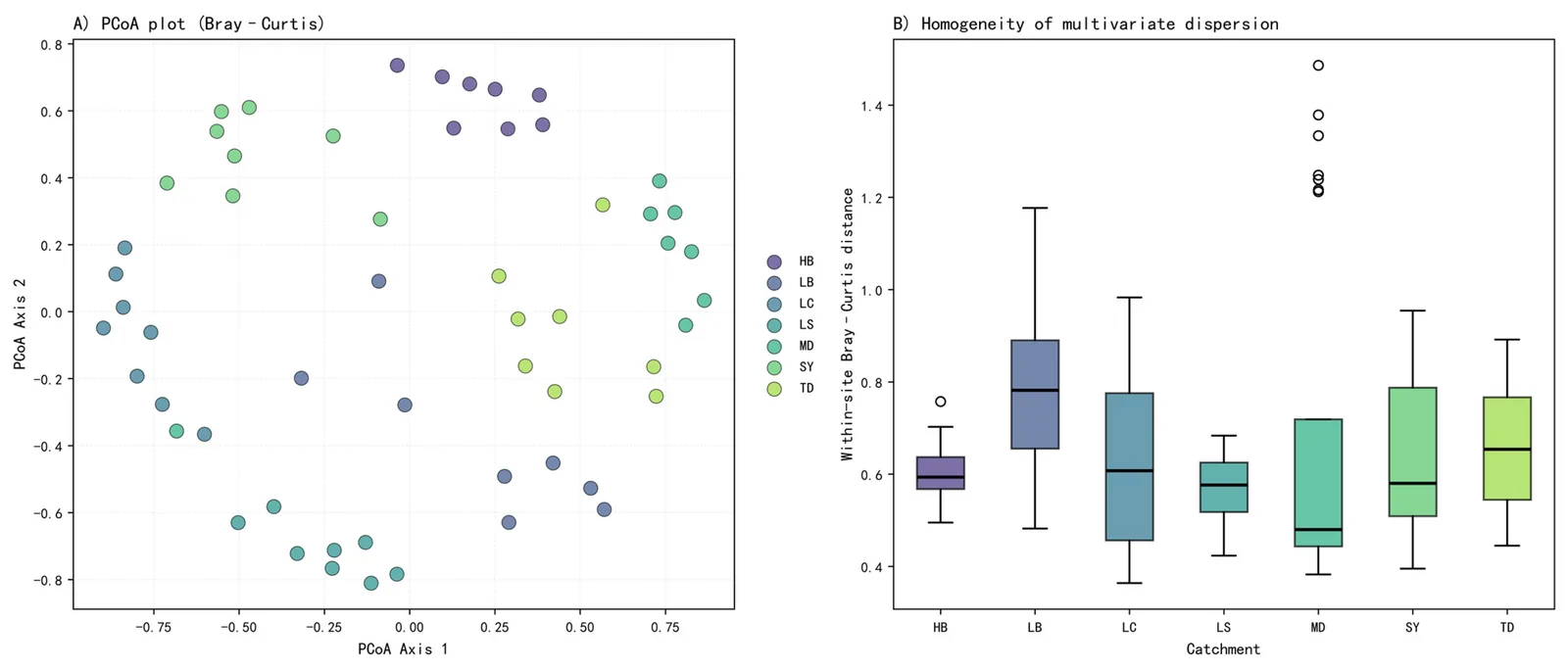
Spatial organization of the wastewater metabolome. (**A**) PCoA plot based on Bray–Curtis distances (*n* = 56 samples from 7 catchments), colored by catchment. (**B**) Homogeneity of multivariate dispersion among catchments (PERMDISP, *p* = 0.082). Boxplots show within-catchment Bray–Curtis distances.

**Figure 2:**
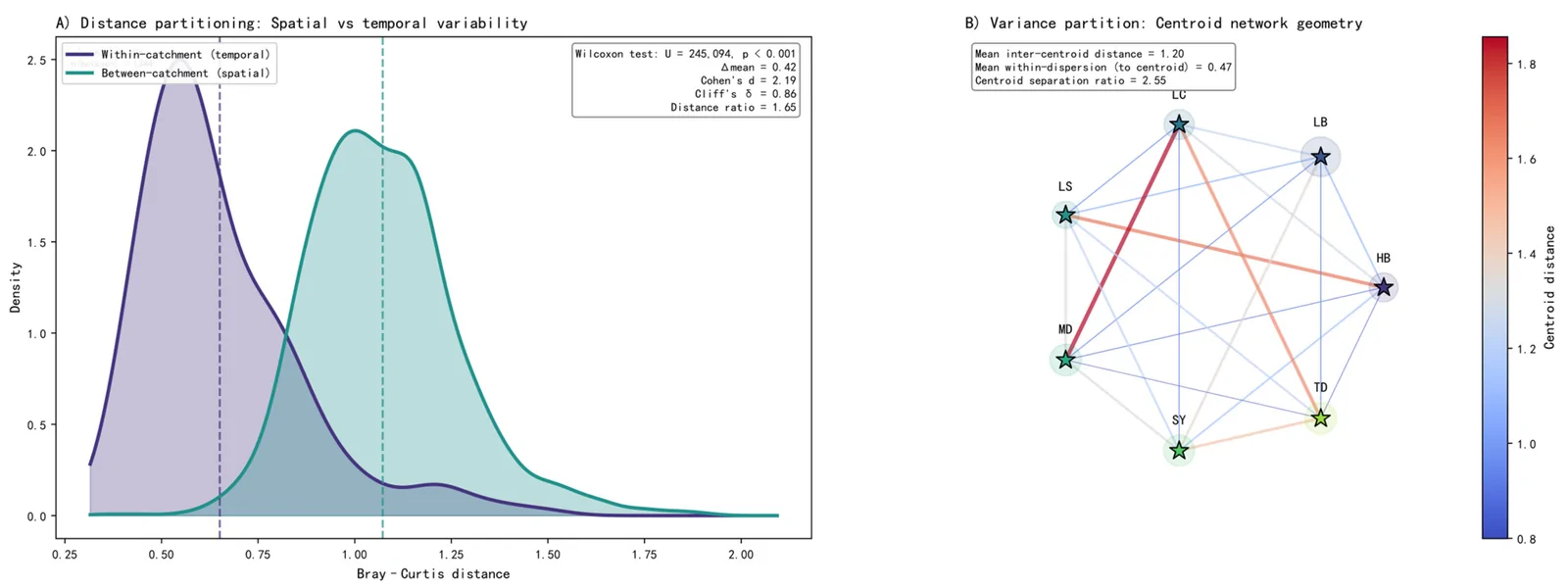
Stability of catchment-specific metabolic configurations. (**A**) Density distribution of Bray–Curtis distances within (temporal variability) and between (spatial structure) catchments across eight sampling days. Within-catchment distances were significantly lower than between-catchment distances (Wilcoxon test, *p* < 0.001; pairwise distance ratio = 1.65). (**B**) Centroid network geometry showing variance partitioning. Stars represent catchment centroids, circles indicate mean sample-to- centroid dispersion radii, and connecting lines show inter-centroid distances (weighted by magnitude). The ratio of mean inter-centroid distance to mean within-dispersion was 2.55, demonstrating that spatial separation exceeded temporal variability.

**Figure 3:**
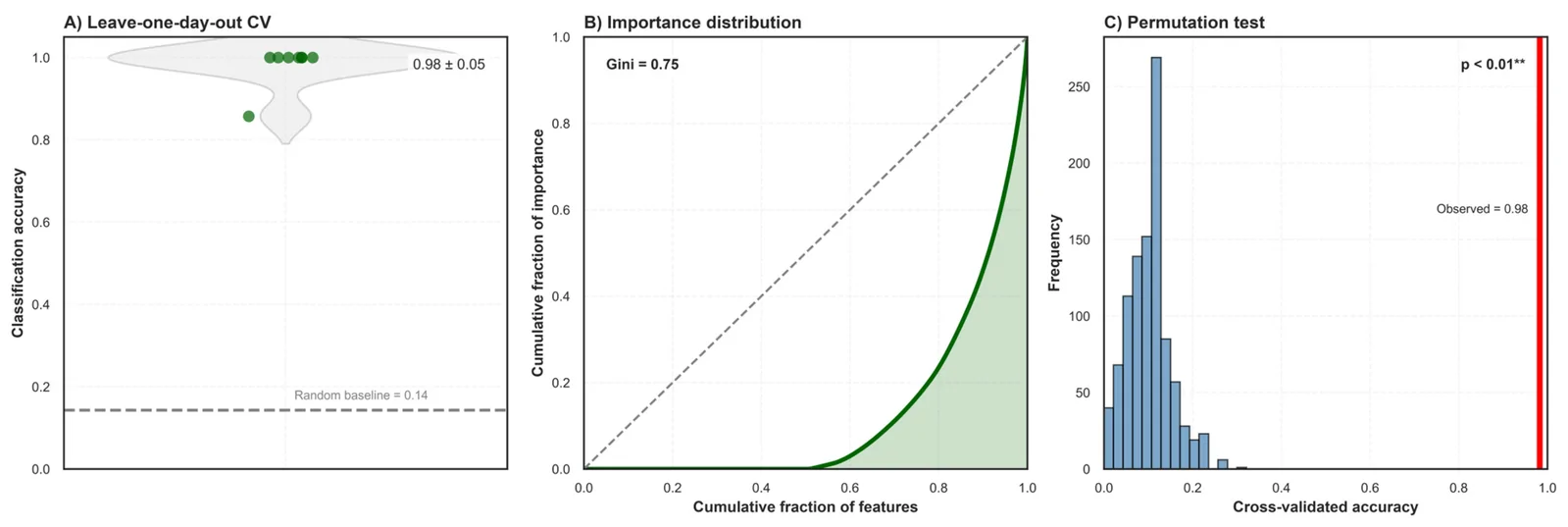
High-dimensional separability of catchments based on metabolomic fingerprints. (**A**) Classification accuracy obtained using random forest models under leave-one-day-out cross-validation (eight folds). Points represent held-out days; the violin plot shows the distribution of accuracies across folds. The dashed horizontal line indicates the random baseline accuracy expected for seven equally represented classes (0.14). Mean ± standard deviation is shown. (**B**) Cumulative distribution of feature importance ranked by mean decrease in Gini impurity. The Lorenz curve illustrates the concentration of predictive importance across metabolites; the dashed diagonal line represents perfect equality. The Gini coefficient quantifies importance concentration. (**C**) Permutation test results (1000 iterations) showing the null distribution of cross-validated classification accuracy under randomized class labels. The red vertical line indicates the observed accuracy. The *p*-value was calculated as *p* = *n*/*B*, where *n* is the number of permuted accuracies greater than or equal to the observed accuracy, and *B* is the total number of permutations (*B* = 1000). **, *p* < 0.01.

**Figure 4:**
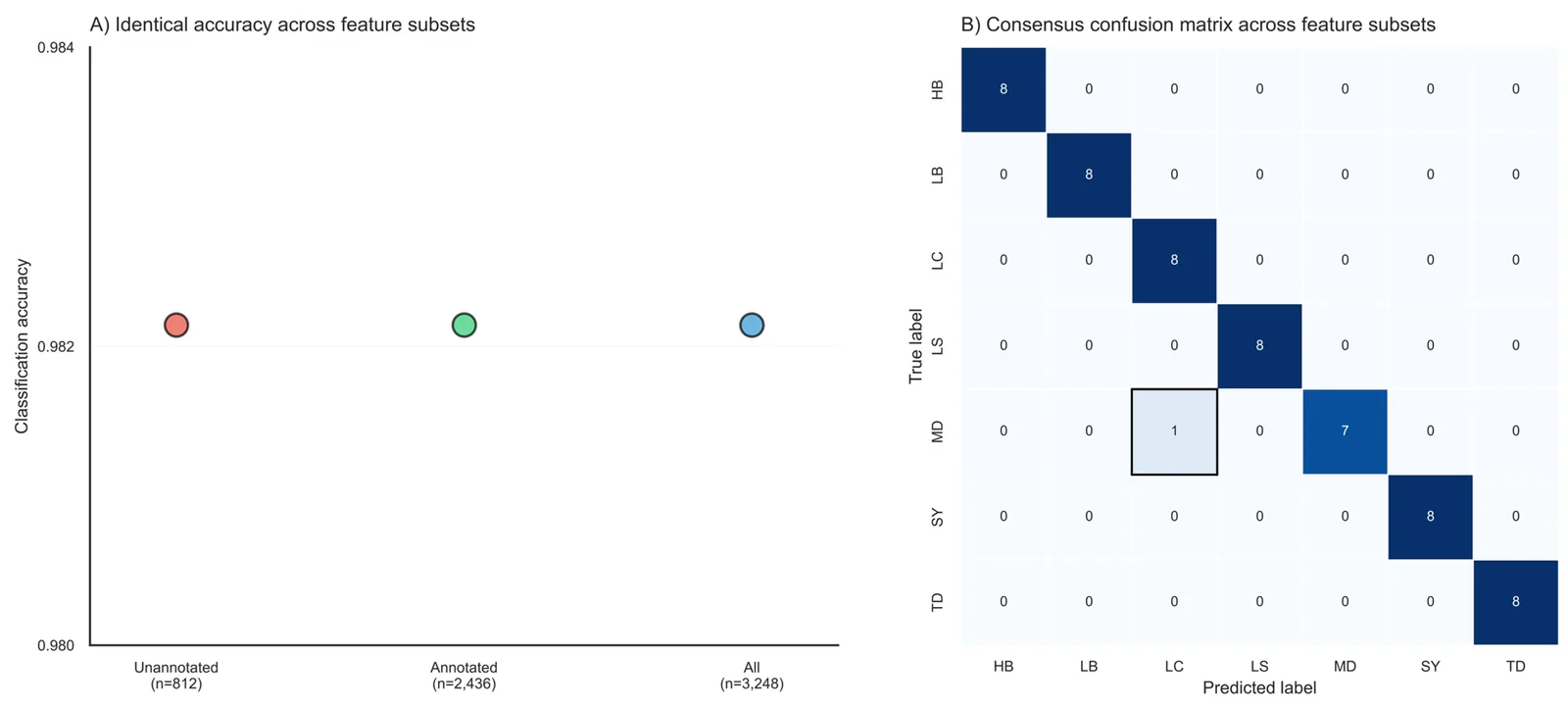
Robust classification performance across feature subsets. (**A**) Classification accuracy obtained using unannotated features (*n* = 812), annotated features (*n* = 2436), and the combined feature set (*n* = 3248). Identical mean accuracy (98.2%) was observed across all feature subsets. (**B**) Consensus confusion matrix aggregated across feature subsets. Values represent the number of samples per class (*n* = 8 per class). A single misclassification (MD predicted as LC) was consistently observed.

**Figure 5:**
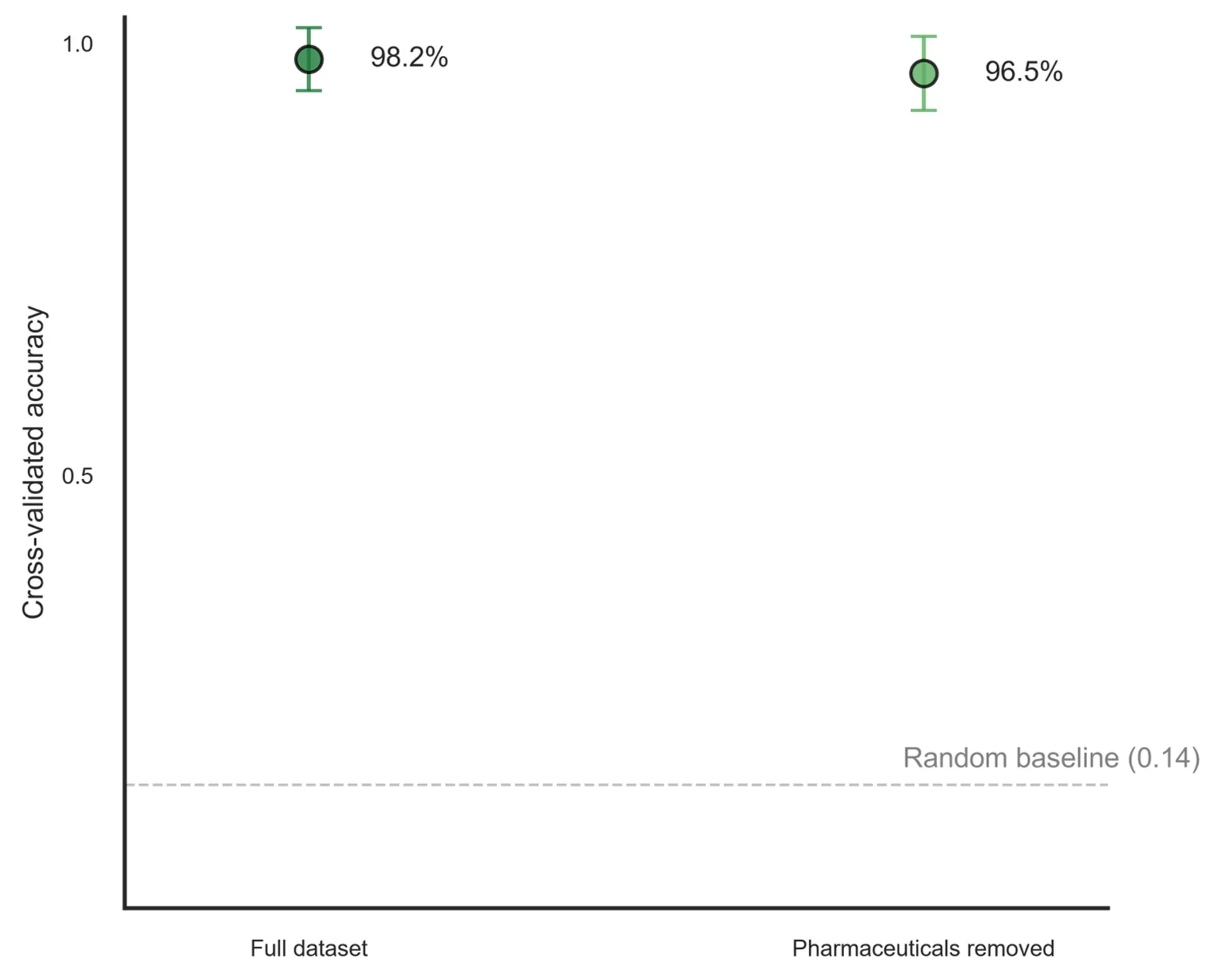
Classification accuracy after removal of annotated pharmaceuticals. Comparison of cross-validated classification accuracy before and after removal of 294 annotated pharmaceutical compounds. Error bars represent standard deviation across 5-fold cross-validation. The high accuracy (96.5%) maintained after pharmaceutical removal indicates that spatial separability was not solely attributable to exogenous pharmaceutical signals. Statistical analysis using the Wilcoxon signed-rank test showed no significant difference between models (*p* = 1.000), confirming that pharmaceutical removal did not systematically impair classification performance. Note: Full dataset: 98.2% ± 3.6%; Pharmaceuticals removed: 96.5% ± 4.3%.

## Data Availability

The raw data supporting the conclusions of this article will be made available by the authors on request.

## Supplementary Materials

The following supporting information can be downloaded at: https://www.mdpi.com/article/10.3390/metabo16100702/s1. Table S1: Basic characteristics of the seven WWTP catchments; Table S2: PERMANOVA. Effect of Catchment on the Response Variable; Table S3: Distance to Group Spatial Median by Catchment; Table S4: Random Forest Model Hyperparameters and Training Configuration; Table S5: Molecular features consistently ranked among the top 100 most important features in ≥70% of the eight leave-one-day-out folds; Table S6: Per-fold classification performance of the Random Forest model under leave-one-day-out (LODO) and leave-one-site-out (LOSO) cross-validation. Table S7: Top 100 Features by Importance. Figure S1: PCA analysis of QC and biological samples.
