# Supplementary material for: Short-Term Stable Catchment-Level Metabolic Structure in Urban Wastewater Revealed by Untargeted Metabolomics
Source: Metabolites. 2026 Sep 22;16(10):702. doi: 10.3390/metabo16100702 (PMC13706761; doi:10.3390/metabo16100702)
Supplement: Supplementary file 1 [file metabolites-16-00702-s001.zip › metabolites-4530176-supplementary.pdf]

Supplementary Materials

Short-Term Stable Catchment-Level Metabolic Structure in Urban Wastewater Revealed by Untargeted Metabolomics

Dandan Li, Yin Wang, Yanfei Shi, Zhen Ma, Yuan Fang, Yan Ma, Bingling Wang*, Haiping Duan*, Kunzheng Lv*

1 Data Preprocessing Workflow

1.1 Ion Mode Processing

├── Positive ion mode (separate)
└── Negative ion mode (separate)

↓

1.2 Normalization

└── Total peak area normalization for each ion mode separately

↓

1.3 Data Integration

└── Combine positive and negative ion mode data

↓

1.4 Software

└── Python (pattern recognition and hierarchical processing)

↓

1.5 Annotation Levels (4-tier system)

├── Level 1: RT + MS1 + MS2 (highest confidence)
├── Level 2: MS1 + MS2
├── Level 3: MS1 + RT
└── Level 4: MS1 only (lowest confidence)

↓

1.6 Statistical Analysis

└── Only top 2 levels (Level 1 and Level 2) retained for downstream analysis

↓

1.7 Quality Control

├── PCA analysis: All experimental and QC samples (after UV processing)
├── Cross-validation: 7-fold
├── QC clustering: Tight clustering in PCA space
├── Reproducibility: Excellent (demonstrated by tight QC clustering)
├── Instrument stability: Monitored throughout
├── Instrument performance: Monitored via QC samples
├── Batch effects: Corrected using ComBat algorithm
├── Reproducibility: CV < 20% for QC samples
├── Method blank: Used to assess background contamination
└── UV processing: Applied to all samples for consistency

2 WWTP catchment characteristics

2.1 Basic characteristics of the seven WWTP catchments

Table S1: Basic characteristics of the seven WWTP catchments

Site	Population served	Daily influent flow (m^3/d)	Predominant wastewater composition
TD	260,000	78,000–92,991	Residential sewage
HB	740,000	155,000–171,000	Residential sewage
LC	1,200,000	251,001–293,000	Residential sewage plus industrial wastewater
LS	460,000	198,900–225,700	Residential sewage
SY	45,000	4,624–5,200	Residential sewage from the area surrounding the Park and green space
MD	1,380,000	129,000–133,000	Residential sewage
LB	1,200,000 [a]	42,800–48,000	Phase IV extension of LC; shares the influent pipeline with the LC [b]

Data source: Qingdao Drainage Operation Service Center.

Note: [a]Serves the same population as the LC plant. [b]Although the two plants receive wastewater from the same pipeline, influent quality may differ slightly between them.

3 Quality Control

3.1 Analytical drift assessment

Figure S1 PCA analysis of QC and biological samples

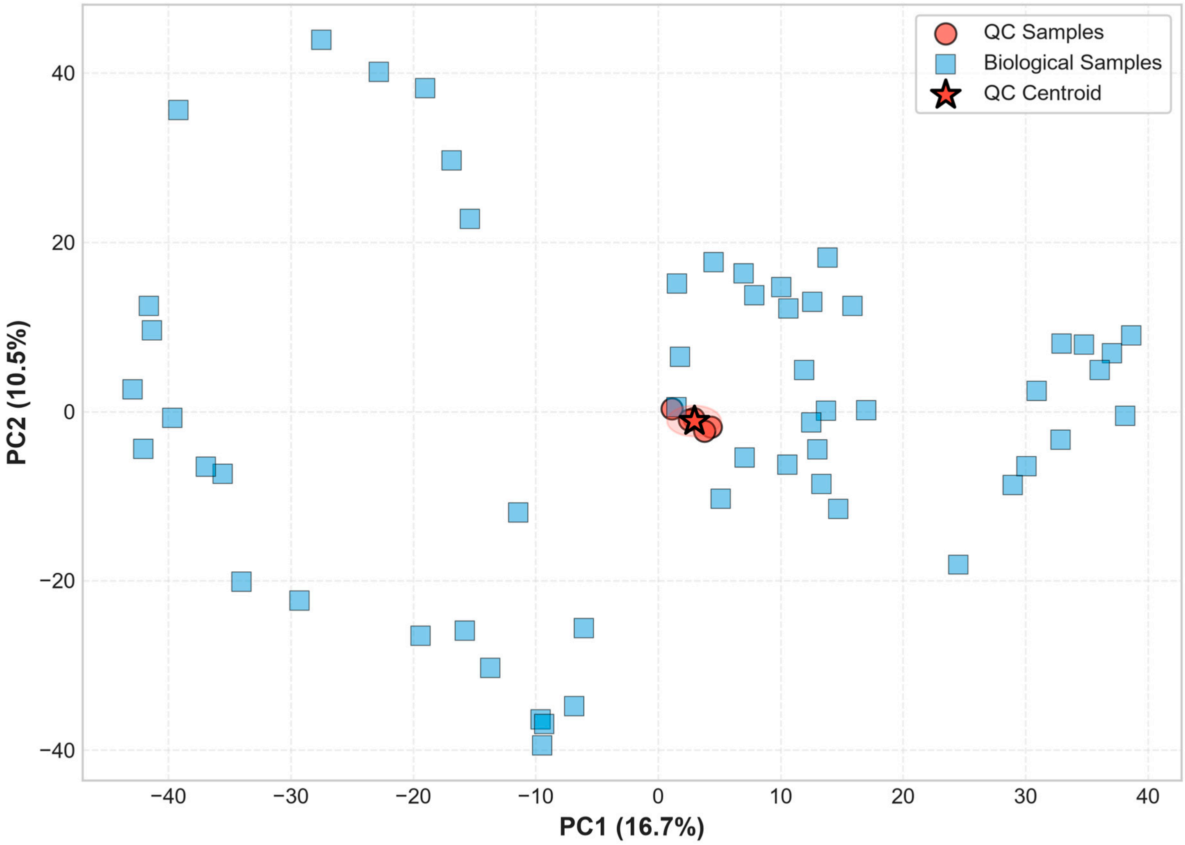

2 PERMANOVA Complete Results

2.1 Analytical parameters

PERMANOVA (Permutational Multivariate Analysis of Variance) was performed to assess significant differences in global metabolomic composition among catchments. The analysis was conducted using the Bray-Curtis distance metric, which is widely used in ecological and metabolomic studies for its effectiveness in capturing compositional differences. To ensure robust statistical inference, a permutation test with 9,999 permutations was employed, providing reliable p-values for hypothesis testing. The significance level was set at $\alpha = 0.05$, consistent with standard statistical practices in metabolomic research. This analytical approach allowed for the assessment of whether catchment identity significantly explained variation in metabolomic profiles.

2.2 Complete ANOVA table

Table S2: PERMANOVA. Effect of Catchment on the Response Variable

**Source of Variation**	**df**	**Sum of Squares (SS)**	**Mean Squares (MS)**	**F-value**	**R^2**	**p-value**
**Catchment**	6	37.432	6.239	13.42	0.62	**< 0.001**
**Residual**	49	22.780	0.465	—	0.38	—
**Total**	55	60.212	—	—	1.00	—

2.3 Key statistics

PERMANOVA analysis revealed highly significant differences in the overall metabolomic composition across different catchments ($F = 13.42$, $df = 6, 49$, $p < 0.001$). Catchment identity explained 62% of the total variance in metabolomic profiles ($R^2 = 0.62$), indicating that it is a key determinant shaping the metabolic landscape.

3 PERMDISP Detailed Results

3.1 Analysis Parameters

The analysis was conducted using the Bray-Curtis dissimilarity metric. The significance of group dispersion (homogeneity of multivariate variances) was assessed using the distance-to-centroid method, with 999 permutations and a significance level (α) of 0.05.

3.2 Main Results

The test for PERMDISP yielded a non-significant result ($F = 1.88$, $df = 6, 49$, $p = 0.082$). This indicates that the observed differences in group centroids from the subsequent PERMANOVA are unlikely to be driven by significant differences in within-group variation (dispersion). The effect size ($\eta^2 = 0.187$) suggests that approximately 18.7% of the variation in distances to the spatial median can be attributed to catchment identity.

3.3 Mean Distance to Centroid by Catchment

Table S3: Distance to Group Spatial Median by Catchment

Catchment	Mean Distance	SD	n
HB	0.433	0.025	8
LB	0.593	0.121	8
LC	0.452	0.072	8
LS	0.405	0.038	8
MD	0.471	0.237	8
SY	0.464	0.109	8
TD	0.475	0.046	8

3.4 Pairwise Comparisons (Tukey HSD)

No significant pairwise differences were detected after multiple testing correction (all $p > 0.05$).

3.5 Interpretation

PERMDISP indicated no significant differences in multivariate dispersion among catchments ($p = 0.082$), suggesting that spatial separation was driven by shifts in centroid location rather than differences in dispersion magnitude.

4 Random Forest Model Parameters

4.1 Model Configuration

Table S4: Random Forest Model Hyperparameters and Training Configuration

Parameter	Value	Description
n_estimators	100	Number of decision trees in the forest.
max_features	sqrt(n_features)	Number of features to consider for the best split at each node; set to the square root of the total number of features.

max_depth	10	Maximum depth of an individual tree.
min_samples_split	2	Minimum number of samples required to split an internal node.
min_samples_leaf	1	Minimum number of samples required to be at a leaf node.
class_weight	'balanced'	Automatically adjusts weights inversely proportional to class frequencies to handle potential class imbalance.
Cross-Validation	Stratified 5-Fold	A 5-fold cross-validation was used, with stratification to preserve the original class distribution in each fold.
random_state	42	Seed for the random number generator to ensure reproducibility.
n_jobs	-1	Utilizes all available processor cores for parallel computation.

4.1Feature Stability

Table S5: Molecular features consistently ranked among the top 100 most important features in ≥70% of the eight leave-one-day-out folds (i.e., present in ≥6 of 8 folds). "Tier" indicates whether the feature remained within the top 20 ("Top20") or only within the top 100 ("Top100") most important features per fold. "Folds ranked" is the number of folds (out of 8) in which the feature appeared among the top-ranked features. "---" in Superclass denotes unannotated features. In total, 42 features were stable within the top 100 (of which 6 within the top 20), and 188 features were stable within the top 10% of ranked features.

No.	Alignment ID	Metabolite name	Adduct ion	Observed m/z	Theoretical m/z	Mass error (ppm)	MS/MS fragment ions, m/z (relative intensity, %)	Superclass	Tier	Folds ranked (of 8)
1	NEG7422	(11E,13Z)-1,7-dioxacyclooctadeca-11,13-diene-2,8-dione/mesuadione	[M-H]-	279.1635	279.1596	+13.67	96.9585 (100.0), 79.9555 (7.2), 235.1703 (6.1), 95.9511 (4.5), 280.0768 (3.7), 235.1338 (2.2), ...	---	Top100	8
2	NEG9550	[3aR-(3aalpha,6alpha,8abeta,9aalpha)]-5-[(Acetyloxy)methyl]-3a,4,6,7,8,8a,9,9a-octahydro-6-hydroperoxy-8a-methyl-3-methylenenaphtho[2,3-b]furan-2(3H)-one	[M-H]-	321.1338	321.1338	-0.07	321.2089 (100.0), 277.1447 (97.9), 233.1534 (77.1), 321.1707 (52.9), 80.9633 (31.6), 165.0176 (30.5), ...	---	Top100	8
3	NEG7665	1-(4-Chlorobenzhydryl)piperazine	[M-H]-	285.1160	285.1159	+0.52	205.1592 (100.0), 285.2044 (63.9), 79.9556 (36.4), 267.1950 (9.5), 67.6279 (8.4), 206.1608 (7.3), ...	---	Top100	8
4	POS8272	3-Hydroxymonoethylglycinexylidide	[M+H]+	223.1438	223.1447	-3.78	126.0911 (100.0), 98.0609 (73.7), 83.0862 (41.8), 178.1223 (38.7), 225.0410 (35.3), 165.0542 (33.1), ...	Organic acids and derivatives	Top20	8
5	NEG2989	4-Chlorophenoxyacetic acid	[M-H]-	185.0000	185.0005	-3.12	126.9933 (100.0), 79.9555 (14.5), 186.0618 (12.1), 185.1170 (11.4), 127.9969 (5.3), 186.1110 (4.2), ...	Benzenoids	Top100	8
6	NEG1378	4-Vinylcyclohexene dioxide	[M-H]-	139.0749	139.0759	-7.51	124.0512 (100.0), 138.8577 (74.5), 68.7422 (17.7), 95.0482 (14.8), 83.0482 (13.0), 55.0173 (12.4), ...	Organoheterocyclic compounds	Top100	8
7	POS15212	Daphnezomine A	[M+H]+	362.2688	362.2695	-1.90	299.2368 (100.0), 344.2607 (64.2), 316.2636 (49.3), 327.2304 (41.8), 281.2265 (41.6), 163.1484 (27.9), ...	Lipids and lipid-like molecules	Top100	8
8	NEG8798	Dillanol	[M-H]-	307.0613	307.0606	+2.15	239.0742 (100.0), 227.0331 (98.6), 229.1337 (30.6), 306.9899 (30.1), 307.1175 (26.0), 240.0805 (18.9), ...	Phenylpropanoids and polyketides	Top100	8
9	POS18044	E-3174	[M+H]+	437.1481	437.1493	-2.69	207.0901 (100.0), 235.0960 (75.2), 180.0797 (24.3), 190.0659 (21.9), 206.0847 (11.5), 203.0573 (9.1), ...	Benzenoids	Top20	8
10	NEG14309	Losartan carboxylic acid	[M-H]-	435.1336	435.1336	-0.11	157.0526 (100.0), 391.1408 (16.1), 363.1366 (4.5), 158.0566 (3.5), 113.9970 (2.4), 392.1479 (2.1), ...	Benzenoids	Top20	8
11	NEG7321	Monooctyl phthalate	[M-H]-	277.1436	277.1440	-1.57	121.0282 (100.0), 122.0308 (4.8), 120.0204 (4.7), 81.9517 (0.9), 219.1746 (0.7), 134.0355 (0.7), ...	Benzenoids	Top20	8
12	POS12088	Norfluoxetine	[M]+	295.1201	295.1184	+5.83	69.0453 (100.0), 296.1825 (54.1), 295.1507 (20.5), 295.1988 (15.0), 99.0811 (7.2), 197.1289 (6.1), ...	Benzenoids	Top100	8
13	NEG5070	Thymol Sulfate	[M-H]-	229.0531	229.0535	-1.33	149.0953 (100.0), 79.9555 (37.1), 229.1433 (30.1), 129.0902 (10.1), 150.0983 (9.2), 211.1335 (8.3), ...	Organic acids and derivatives	Top20	8
14	POS3181	2-(cyclopent-1-en-1-yl)propanoic acid	[M+H]+	141.0911	141.0916	-3.29	95.0857 (100.0), 85.0652 (39.1), 123.0805 (33.6), 67.0548 (28.3), 141.0540 (16.6), 85.0288 (15.2), ...	---	Top20	8
15	POS3835	2-Acetyl-3-ethylidene-3,4,5,6-tetrahydropyridine	[M+H]+	152.1068	152.1075	-4.99	153.0656 (100.0), 154.0491 (38.6), 152.0558 (26.7), 121.0647 (2.9), 153.0403 (2.8), 136.0394 (2.5), ...	Organoheterocyclic compounds	Top100	8
16	POS14006	NCGC00380664-01!4-[(E)-3-hydroxy-	[M+H]+	334.2735	334.2746	-3.30	91.0548 (100.0), 242.2107 (62.6), 58.0660 (31.8), 243.2149 (5.9),	Benzenoids	Top100	8

		8,10-dimethyl-2-(methylamino)dodec-6-enyl]phenol [IIN-based: Match]					219.1742 (5.7), 335.1856 (5.2), ...			
17	POS13932	Piptamine(antibiotic)	[M+H]+	332.3301	332.3317	-5.04	91.0543 (100.0), 240.2691 (44.6), 58.0658 (35.9), 241.2719 (5.1), 92.0579 (4.5), 59.0693 (0.6), ...	Benzenoids	Top100	8
18	POS16454	(1R,3aS,3bS,5aS,7aS,11aR,11bS,11cS,13aR)-hexadecahydro-1-ethenyl-11b,13a-dimethyl-7H-cyclopenta[7,8]phenanthro[4,3-e][1,3,4]oxadithiin-7-one/krempene A	[M+H]+	393.1915	393.1922	-1.85	313.2344 (100.0), 311.2617 (56.6), 357.2671 (49.1), 375.2770 (23.9), 376.2568 (22.2), 339.2577 (16.7), ...	---	Top100	7
19	NEG7496	2,8-Dihydroxy-1,7-dimethyl-6-vinyl-10,11-dihydrodibenz[b,f]oxepin	[M-H]-	281.1209	281.1178	+11.17	96.9579 (100.0), 281.0444 (88.2), 281.1740 (81.5), 263.1651 (54.2), 237.0582 (45.1), 238.0651 (21.5), ...	Organoheterocyclic compounds	Top100	7
20	NEG9235	8-[(acetyloxy)methyl]-6,9-dihydro-5-hydroxy-2-methyl-4H-pyrano[3,2-h][1]benzoxepin-4-one/prenyletin/Ptaeroxylinolacetat	[M-H]-	315.0909	315.0869	+12.94	255.0682 (100.0), 79.9558 (43.6), 315.1799 (38.4), 253.0894 (33.5), 73.0278 (23.7), 315.1483 (20.2), ...	---	Top100	7
21	NEG7499	Carolenalol	[M-H]-	281.1391	281.1389	+0.61	237.1474 (100.0), 171.1162 (64.7), 123.0794 (45.2), 201.1279 (28.1), 189.1272 (25.1), 207.1379 (16.8), ...	Organoheterocyclic compounds	Top100	7
22	POS4304	Cleroindicin B	[M+H]+	159.1016	159.1021	-3.14	99.0809 (100.0), 81.0707 (29.0), 160.0756 (5.7), 117.9597 (5.6), 128.9508 (5.4), 131.1178 (5.3), ...	Organic oxygen compounds	Top100	7
23	NEG3486	Ficusic acid	[M-H]-	195.1018	195.1021	-1.53	160.8400 (100.0), 195.8089 (99.9), 151.1113 (27.3), 95.0484 (10.2), 153.0285 (6.4), 93.0324 (3.5), ...	Organoheterocyclic compounds	Top100	7
24	NEG5854	Sesquiterpenes	[M-H]-	245.1210	245.1178	+13.26	79.9554 (100.0), 246.0347 (16.7), 245.0676 (13.1), 96.9583 (12.8), 246.0750 (10.9), 80.9637 (4.4), ...	Lipids and lipid-like molecules	Top100	7
25	POS16887	Sicanatriol 7beta,18-diacetate	[M+H]+	405.2604	405.2641	-9.18	293.1363 (100.0), 89.0603 (33.8), 171.0618 (21.7), 71.0863 (18.5), 127.0365 (15.8), 57.0711 (13.4), ...	Lipids and lipid-like molecules	Top100	7
26	NEG2942	Stagonolide F	[M-H]-	183.1013	183.1021	-4.31	183.0045 (100.0), 94.9152 (20.3), 138.9048 (16.7), 153.0056 (8.5), 184.0059 (5.3), 123.0070 (5.0), ...	---	Top100	7
27	NEG11414	Turbomycin A	[M-H]-	358.1324	358.1344	-5.68	183.0114 (100.0), 197.0258 (57.0), 269.0850 (40.7), 198.0345 (37.5), 255.0679 (35.6), 283.1020 (34.9), ...	---	Top100	7
28	POS18336	7-adamantanyl-1,3-dimethyl-8-(2-pyridylmethyl)-1,3,5-trihydro-4-imidazolino[1, 2-h]purine-2,4-dione	[M+H]+	445.2342	445.2352	-2.20	207.0905 (100.0), 211.1427 (19.3), 208.0947 (7.0), 417.2230 (5.7), 402.2196 (2.2), 428.3192 (1.9), ...	Organoheterocyclic compounds	Top100	7
29	NEG7395	SCHEMBL3181204	[M-H]-	279.0305	279.0293	+4.20	212.0003 (100.0), 184.0182 (84.1), 225.0187 (83.7), 183.0111 (60.9), 185.0263 (23.0), 151.0375 (20.0), ...	---	Top100	7
30	POS7600	(3E,5E)-trideca-3,5-dienoic acid	[M+H]+	211.1689	211.1698	-4.29	109.1011 (100.0), 135.1171 (81.6), 95.0857 (36.3), 212.1995 (36.0), 175.1482 (29.0), 107.0856 (27.8), ...	Lipids and lipid-like molecules	Top100	6
31	NEG12513	16beta-Hydroxydehydroepiandrosterone sulfate	[M-H]-	383.1533	383.1528	+1.27	96.9581 (100.0), 80.9637 (6.1), 69.1501 (3.7), 98.9535 (3.2), 79.9554 (1.4), 255.1489 (1.2), ...	Lipids and lipid-like molecules	Top100	6
32	NEG2996	2-hydroxy-3-(3-oxoprop-1-enyl)but-2-enedioic acid	[M-H]-	185.0115	185.0086	+15.88	96.9584 (100.0), 57.9740 (23.4), 184.8598 (15.9), 185.8430 (14.5), 122.9734 (13.2), 186.1123 (10.3), ...	Lipids and lipid-like molecules	Top100	6
33	NEG1927	3-Methyladipic acid	[M-H]-	159.0650	159.0657	-4.80	97.0644 (100.0), 115.0746 (93.9), 141.0549 (4.4), 98.0679 (4.3), 116.0786 (4.2), 158.8448 (4.2), ...	Lipids and lipid-like molecules	Top100	6
34	NEG13220	5-(4-methoxyanilino)-5-oxo-3-(3,4,5-trimethoxyphenyl)pentanoic acid	[M-H]-	402.1589	402.1553	+9.03	186.1485 (100.0), 79.9557 (26.5), 168.1370 (11.5), 80.9632 (8.9), 96.9586 (8.4), 187.1523 (5.9), ...	---	Top100	6

35	NEG9734	6beta-acetoxy-7beta,8beta-dihydroxy-11alphaH-eremophilan-(12,8alpha)-olide	[M-H]-	325.1690	325.1651	+12.08	96.9583 (100.0), 265.1433 (22.0), 195.1740 (20.0), 307.1898 (17.7), 209.1170 (12.3), 122.9746 (7.6), ...	---	Top100	6
36	NEG7830	Apigeninidin	[M-H]-	289.0267	289.0268	-0.32	253.0514 (100.0), 254.0546 (10.6), 289.1050 (3.4), 289.1469 (2.7), 129.0902 (2.0), 79.9556 (1.7), ...	Phenylpropanoids and polyketides	Top100	6
37	POS12737	Clopidogrel carboxylic acid	[M+H]+	308.0506	308.0512	-1.86	198.0320 (100.0), 152.0256 (29.1), 169.0053 (15.6), 170.0373 (12.8), 199.0351 (7.8), 309.1322 (4.4), ...	Organic acids and derivatives	Top100	6
38	NEG6708	Hulupinic acid	[M-H]-	263.1320	263.1283	+13.78	96.9586 (100.0), 244.1319 (14.0), 79.9552 (7.8), 148.9177 (7.5), 193.0706 (7.1), 194.0770 (6.7), ...	Organic acids and derivatives	Top100	6
39	NEG7661	Isosakuranetin	[M-H]-	285.0795	285.0763	+11.23	183.0109 (100.0), 79.9555 (53.8), 267.0689 (44.1), 68.3708 (29.8), 241.0894 (26.3), 184.0181 (6.3), ...	Phenylpropanoids and polyketides	Top100	6
40	NEG12945	Sucralose	[M-H]-	395.0063	395.0067	-1.10	359.0313 (100.0), 395.1338 (37.6), 321.1328 (23.7), 87.0070 (14.8), 143.0330 (14.2), 72.9911 (13.4), ...	Organic oxygen compounds	Top100	6
41	POS15761	Azithromycin	[M+2H]2+	375.2602	375.2621	-5.14	83.0496 (100.0), 158.1172 (26.4), 116.1070 (18.9), 115.0757 (7.0), 116.0703 (6.3), 72.0816 (5.4), ...	Organic oxygen compounds	Top100	6
42	POS10659	Oxprenolol	[M+H]+	266.1746	266.1756	-3.98	91.0547 (100.0), 74.0606 (47.1), 103.0391 (36.2), 71.0498 (31.9), 166.0866 (29.2), 174.1128 (21.5), ...	Benzenoids	Top100	6

Note: Putative annotations were obtained by matching the observed precursor m/z against the theoretical m/z of candidate molecular formulae in HMDB, MassBank, GNPS and an in-house spectral library, and were retained only when the accurate-mass match was combined with an MS/MS spectral match (<0.01 Da) (main text, Section 2.4; SI, Section 1.5). To make the mass accuracy of each individual annotation transparent, the assigned adduct ion, the observed and theoretical m/z and the resulting relative mass error (ppm) are reported for every compound. The 20 ppm window was used only as the initial database search window, and the actual mass errors of the retained annotations were much smaller (median |error| 3.9 ppm; 64% within ±5 ppm and 81% within ±10 ppm; maximum 15.9 ppm). “Theoretical m/z” is calculated from the molecular formula and the assigned adduct ion; “Mass error” is the relative deviation (ppm) between the observed and the theoretical m/z. “MS/MS fragment ions” lists up to the 6 most abundant product ions of the HCD MS/MS spectrum (m/z with relative intensity, %), with the surviving precursor ion and its ^{13}C isotopologue excluded; “...” indicates additional lower-abundance fragments and “n.d.” indicates that no MS/MS spectrum was recorded. All listed annotations correspond to putatively identified compounds (MSI Level 2); no authentic-standard-confirmed (MSI Level 1) assignments are claimed.

4.2 Cross-Validation Parameters

The model's performance was evaluated using a Stratified 5-fold cross-validation scheme. This method ensures that each fold maintains the same proportion of samples from each catchment (site) as the original dataset. The data was shuffled before splitting to randomize sample order, and a fixed random seed (42) was used to guarantee the reproducibility of the fold assignments.

Table S6: Per-fold classification performance of the Random Forest model under leave-one-day-out (LODO) and leave-one-site-out (LOSO) cross-validation.

(A) Leave-one-day-out (temporal validation) — each fold holds out the seven samples collected on a single sampling day; accuracy is the fraction of held-out samples assigned to the correct catchment.

**Held-out day**	**Correct / Total**	**Accuracy**
Day 1	7/7	1.000
Day 2	7/7	1.000
Day 3	7/7	1.000
Day 4	7/7	1.000
Day 5	7/7	1.000
Day 6	6/7	0.857
Day 7	7/7	1.000
Day 8	7/7	1.000
**Overall**	**55/56**	**0.982 ± 0.047**

(B) Leave-one-site-out (spatial validation) — each fold holds out all eight samples of one catchment.

**Held-out catchment (code)**	**Correct / Total**	**Accuracy**
HB (Haibohe)	0/8	0.000
LB (Licunhe North Bank)	0/8	0.000

Held-out catchment (code)	Correct / Total	Accuracy
LC (Licunhe)	0/8	0.000
LS (Loushanhe)	0/8	0.000
MD (Maidao)	0/8	0.000
SY (Shiyuanhui)	0/8	0.000
TD (Tuandao)	0/8	0.000
**Overall**	**0/56**	**0.000**

Notes:

- Classifier: random forest with 500 trees, max_depth = 10, random_state = 42; features z-score standardized (3,248 features); all classes balanced (8 samples per catchment).
- Group-aware splitting (samples nested within day/site) prevents data leakage between training and test folds.
- Random-chance accuracy for the seven-catchment classification task is $1/7 \approx 0.143$; LODO accuracy significantly exceeded chance (permutation test, $p \leq 0.001$).
- LOSO accuracy of 0% across all catchments indicates that catchment assignment relied on catchment-specific combinatorial signal that does not generalize across catchments.

4.3 Class Balance Information

The dataset consists of 56 total samples, evenly distributed across the 7 catchment classes. Each class contains exactly 8 samples, resulting in a perfectly balanced dataset. Consequently, no artificial class weighting was necessary during model training, as all classes inherently have equal representation (a weight of 1.0).

4.4 Feature Scaling

Prior to model training, all features (metabolomic variables) were standardized using z-score normalization (StandardScaler). This preprocessing step centers the data by subtracting the mean and scales it to unit variance. This ensures that all features contribute equally to the model's distance calculations and prevents features with larger numerical ranges from dominating the analysis.

5 Variable Importance (Top 100)

5.1 Feature Importance Calculation

Feature importance was quantified using the Mean Decrease in Impurity (MDI), also known as Gini importance. This method measures the total reduction in node impurity (Gini index) achieved by a feature across all splits in all decision trees within the forest. The importance

for each feature was calculated by averaging its total impurity decrease across all 100 trees. The reported values are the raw, non-normalized importance scores, providing a direct measure of each feature's relative contribution to the model's predictive accuracy.

5.2 Top 100 Features by Importance

Table S7: Top 100 Features by Importance

Rank	Feature ID	Observed m/z	RT (min)	Adduct ion	Theoretical m/z	Mass error (ppm)	MS/MS fragment ions, m/z (relative intensity, %)	Importance	Annotation
1	NEG14309	435.1336	6.83	[M-H]-	435.1336	-0.11	157.0526 (100.0), 391.1408 (16.1), 363.1366 (4.5), 158.0566 (3.5), 113.9970 (2.4), 392.1479 (2.1), ...	0.007911	Losartan carboxylic acid
2	NEG16299	507.1091	5.79	[M-H]-	507.1080	+2.18	253.0514 (100.0), 254.0548 (7.9), 67.7773 (5.6), 507.3019 (0.8), 439.2551 (0.7), 507.1718 (0.7), ...	0.007084	CHEMBL3104732
3	NEG5854	245.1210	6.47	[M-H]-	245.1178	+13.26	79.9554 (100.0), 246.0347 (16.7), 245.0676 (13.1), 96.9583 (12.8), 246.0750 (10.9), 80.9637 (4.4), ...	0.006858	Sesquiterpenes
4	POS9859	251.1270	7.10	[M+H]+	251.1283	-5.43	195.0659 (100.0), 177.0543 (66.3), 153.0547 (23.5), 196.0673 (8.3), 178.0573 (6.1), 233.1159 (3.6), ...	0.006739	Methylripariochromene A
5	NEG7124	273.0439	4.55	[M-H]-	273.0399	+14.42	79.9554 (100.0), 229.0514 (64.1), 149.0952 (64.1), 229.0967 (13.0), 273.0933 (7.0), 193.0501 (6.7), ...	0.006408	Myxotrichin D
6	NEG4091	209.0443	4.89	[M-H]-	209.0450	-3.48	121.0279 (100.0), 209.0788 (9.0), 91.0534 (7.5), 122.0316 (5.6), 165.0550 (3.3), 151.0389 (2.4), ...	0.006394	Vanilpyruvic acid
7	POS13232	318.1088	6.83	[M+Na]+	318.1106	-5.60	319.2396 (100.0), 209.1318 (36.5), 318.3011 (26.0), 319.1855 (22.8), 219.1743 (12.0), 256.2613 (10.2), ...	0.005967	6-ethoxy-4-hydroxy-3-benzylhydroquinolin-2-one
8	POS15324	365.2068	5.57	[M+Na]+	365.2052	+4.40	172.1320 (100.0), 305.1855 (22.0), 365.1445 (19.8), 269.1887 (11.4), 259.1809 (8.3), 173.1371 (6.1), ...	0.005913	Aloxistatin
9	NEG2989	185.0000	6.18	[M-H]-	185.0005	-3.12	126.9933 (100.0), 79.9555 (14.5), 186.0618 (12.1), 185.1170 (11.4), 127.9969 (5.3), 186.1110 (4.2), ...	0.005834	4-Chlorophenoxyacetic acid
10	NEG12945	395.0063	4.56	[M-H]-	395.0067	-1.10	359.0313 (100.0), 395.1338 (37.6), 321.1328 (23.7), 87.0070 (14.8), 143.0330 (14.2), 72.9911 (13.4), ...	0.005755	Sucralose
11	NEG7496	281.1209	6.76	[M-H]-	281.1178	+11.17	96.9579 (100.0), 281.0444 (88.2), 281.1740 (81.5), 263.1651 (54.2), 237.0582 (45.1), 238.0651 (21.5), ...	0.005697	2,8-Dihydroxy-1,7-dimethyl-6-vinyl-10,11-dihydrodibenz[b,f]oxepin
12	NEG12358	379.2480	5.73	[M-H]-	379.2484	-1.05	307.1739 (100.0), 379.1846 (86.5), 321.1916 (44.3), 343.2124 (41.4), 297.1146 (39.2), 335.1837 (35.7), ...	0.005648	Membrenone A
13	NEG1021	128.0339	2.15	[M-H]-	128.0348	-7.01	68.0131 (100.0), 82.0278 (20.4), 68.0173 (19.4), 84.0434 (13.4), 68.1868 (11.8), 100.7898 (11.6), ...	0.005626	Pyroglutamic acid
14	POS8219	222.1481	4.50	[M+H]+	222.1494	-5.73	204.1376 (100.0), 147.0438 (27.3), 225.0413 (15.9), 205.1427 (11.1), 176.1437 (8.8), 222.1118 (3.6), ...	0.005561	ZINC31165273
15	POS9753	249.1478	7.26	[M+H]+	249.1491	-5.18	249.1827 (100.0), 175.1483 (59.6), 107.0858 (44.5), 95.0858 (41.1), 121.1015 (39.7), 250.1780 (34.7), ...	0.005533	4-(4,8-Dimethyl-2,4,6-nonatrienyl)-2,6-dioxabicyclo[3.1.0]hexan-3-one
16	POS12737	308.0506	5.10	[M+H]+	308.0512	-1.86	198.0320 (100.0), 152.0256 (29.1), 169.0053 (15.6), 170.0373 (12.8), 199.0351 (7.8), 309.1322 (4.4), ...	0.005529	Clopidogrel carboxylic acid
17	POS10205	258.1247	6.21	[M+2H]2+	258.1263	-5.94	211.0754 (100.0), 212.0794 (13.2), 183.0802 (1.7), 165.0698 (1.5), 223.1702 (1.3), 155.0857 (1.0), ...	0.005337	Telmisartan
18	NEG7422	279.1635	7.50	[M-H]-	279.1596	+13.67	96.9585 (100.0), 79.9555 (7.2), 235.1703 (6.1), 95.9511 (4.5), 280.0768 (3.7), 235.1338 (2.2), ...	0.005293	(11E,13Z)-1,7-dioxacyclooctadeca-11,13-diene-2,8-dione|mesuadione
19	POS18044	437.1481	6.84	[M+H]+	437.1493	-2.69	207.0901 (100.0), 235.0960 (75.2), 180.0797 (24.3), 190.0659 (21.9), 206.0847	0.005250	E-3174

							(11.5), 203.0573 (9.1), ...		
20	NEG15786	485.2246	4.63	[M-H]-	485.2175	+14.50	427.2151 (100.0), 101.0215 (16.6), 383.1943 (15.2), 428.2216 (10.1), 72.9903 (8.7), 75.0058 (6.9), ...	0.005149	Domesticulide A
21	NEG7665	285.1160	7.22	[M-H]-	285.1159	+0.52	205.1592 (100.0), 285.2044 (63.9), 79.9556 (36.4), 267.1950 (9.5), 67.6279 (8.4), 206.1608 (7.3), ...	0.005098	1-(4-Chlorobenzhydryl)piperazine
22	NEG9293	316.1585	6.46	[M-H]-	316.1562	+7.24	79.9553 (100.0), 316.6373 (27.6), 138.0216 (23.2), 315.6292 (16.7), 106.9790 (8.6), 205.0408 (7.5), ...	0.004956	1-NAPHTHYL PP1
23	NEG8986	310.0041	6.78	[M-H]-	310.0038	+1.21	266.0133 (100.0), 311.2216 (45.3), 292.1977 (17.9), 310.2086 (16.2), 230.0368 (14.6), 311.1851 (13.7), ...	0.004819	4'-Hydroxydiclofenac
24	NEG5070	229.0531	5.90	[M-H]-	229.0535	-1.33	149.0953 (100.0), 79.9555 (37.1), 229.1433 (30.1), 129.0902 (10.1), 150.0983 (9.2), 211.1335 (8.3), ...	0.004719	Thymol Sulfate
25	POS16875	405.1717	4.62	[M+H]+	405.1774	-14.19	347.1694 (100.0), 387.1625 (23.2), 89.0604 (21.2), 86.0972 (21.0), 70.0658 (18.5), 319.1379 (18.2), ...	0.004532	Glu Ala Trp
26	NEG12262	377.1969	7.08	[M-H]-	377.1964	+1.21	263.1289 (100.0), 377.1102 (86.8), 96.9588 (70.4), 317.1753 (56.3), 223.1331 (56.1), 333.2039 (49.9), ...	0.004483	Nigakilactone A
27	POS3181	141.0911	4.81	[M+H]+	141.0916	-3.29	95.0857 (100.0), 85.0652 (39.1), 123.0805 (33.6), 67.0548 (28.3), 141.0540 (16.6), 85.0288 (15.2), ...	0.004410	2-(cyclopent-1-en-1-yl)propanoic acid
28	NEG15255	465.3207	7.12	[M-H]-	465.3216	-2.01	401.3048 (100.0), 146.9366 (41.1), 78.9574 (40.3), 190.9278 (34.8), 173.9249 (28.5), 421.2964 (28.1), ...	0.004370	TetraHCA
29	POS10588	265.1173	4.44	[M+H]+	265.1188	-5.63	130.0494 (100.0), 84.0447 (15.7), 136.0755 (14.8), 91.0548 (14.6), 147.0760 (13.7), 129.0661 (13.3), ...	0.004360	Phenylacetylglutamine
30	NEG2942	183.1013	6.25	[M-H]-	183.1021	-4.31	183.0045 (100.0), 94.9152 (20.3), 138.9048 (16.7), 153.0056 (8.5), 184.0059 (5.3), 123.0070 (5.0), ...	0.004300	Stagonolide F
31	NEG7661	285.0795	5.30	[M-H]-	285.0763	+11.23	183.0109 (100.0), 79.9555 (53.8), 267.0689 (44.1), 68.3708 (29.8), 241.0894 (26.3), 184.0181 (6.3), ...	0.004287	Isosakuranetin
32	POS10395	261.1301	3.85	[M+H]+	261.1352	-19.46	86.0970 (100.0), 197.1286 (63.5), 132.1018 (54.6), 243.1324 (54.2), 84.0447 (41.7), 70.0657 (31.4), ...	0.004264	Diaveridine
33	NEG1927	159.0650	4.40	[M-H]-	159.0657	-4.80	97.0644 (100.0), 115.0746 (93.9), 141.0549 (4.4), 98.0679 (4.3), 116.0786 (4.2), 158.8448 (4.2), ...	0.004218	3-Methyladipic acid
34	POS4304	159.1016	5.39	[M+H]+	159.1021	-3.14	99.0809 (100.0), 81.0707 (29.0), 160.0756 (5.7), 117.9597 (5.6), 128.9508 (5.4), 131.1178 (5.3), ...	0.004148	Cleroindicin B
35	POS10998	273.1303	4.73	[M+H]+	273.1338	-12.83	255.1133 (100.0), 114.0915 (52.7), 86.0969 (51.1), 201.0661 (44.4), 274.1777 (44.3), 84.0812 (37.5), ...	0.004125	UXHONHDKVUEWMU-UHFFFAOYSA-
36	POS12378	301.0745	6.92	[M+H]+	301.0744	+0.26	301.2151 (100.0), 165.0906 (78.9), 301.1804 (51.6), 187.1481 (32.5), 283.1687 (32.4), 95.0858 (21.6), ...	0.004090	Clobazam
37	NEG4752	223.0951	5.79	[M-H]-	223.0970	-8.85	179.1068 (100.0), 224.0619 (15.7), 135.0805 (12.3), 59.0122 (9.3), 195.0992 (9.2), 180.1108 (8.2), ...	0.003947	Olivetolic acid
38	POS12004	293.2109	7.12	[M+H]+	293.2117	-2.69	275.1980 (100.0), 294.2390 (80.4), 107.0859 (45.4), 81.0703 (35.6), 95.0860 (33.1), 163.1110 (29.3), ...	0.003939	(2'E,4'Z,7'Z,8E)-Colnelenic acid
39	POS5599	180.1259	4.88	[M+H]+	180.1266	-3.63	135.0679 (100.0), 153.0781 (12.3), 136.0715 (2.5), 152.0949 (1.3), 137.0838 (1.2), 181.0722 (0.6), ...	0.003939	Hexamethylphosphoramide
40	POS10231	258.2421	8.00	[M+H]+	258.2433	-4.66	76.0762 (100.0), 58.0660 (30.0), 227.2016 (7.4), 57.0706 (4.6), 240.2313 (3.4), 71.0862 (3.2), ...	0.003910	Lauryl isopropanolamide

41	NEG12259	377.1964	6.84	[M-H]-	377.1964	-0.09	195.0656 (100.0), 377.1116 (78.5), 275.1641 (69.8), 267.1236 (68.2), 233.1161 (57.5), 251.1276 (47.4), ...	0.003907	bicycloalternarene C
42	POS14006	334.2735	6.22	[M+H]+	334.2746	-3.30	91.0548 (100.0), 242.2107 (62.6), 58.0660 (31.8), 243.2149 (5.9), 219.1742 (5.7), 335.1856 (5.2), ...	0.003839	NCGC00380664-01!4-[(E)-3-hydroxy-8,10-dimethyl-2-(methylamino)dodec-6-enyl]phenol [IIN-based: Match]
43	NEG16891	531.2985	6.39	[M-H]-	531.2992	-1.21	96.9585 (100.0), 369.1708 (68.8), 464.2982 (41.5), 67.6659 (36.4), 327.1648 (32.8), 532.1386 (30.6), ...	0.003798	5b-Cyprinol sulfate
44	POS17194	414.1471	5.10	[M+Na]+	414.1463	+1.89	221.1076 (100.0), 366.1793 (34.5), 269.0756 (15.9), 414.2462 (14.6), 103.0392 (14.4), 89.0603 (11.8), ...	0.003776	(S)-methyl 2-(2-(3-isopropyl-4-oxo-3,4-dihydrophthalazin-1-yl)acetamido)-4-(methylthio)butanoate
45	NEG12513	383.1533	5.48	[M-H]-	383.1528	+1.27	96.9581 (100.0), 80.9637 (6.1), 69.1501 (3.7), 98.9535 (3.2), 79.9554 (1.4), 255.1489 (1.2), ...	0.003754	16beta-Hydroxydehydroepiandrosterone sulfate
46	NEG6617	261.1343	4.83	[M-H]-	261.1338	+1.67	201.1117 (100.0), 225.1117 (29.0), 243.1219 (25.8), 59.0121 (11.1), 199.1330 (9.5), 202.1163 (8.9), ...	0.003749	Triethylene glycol diglycidyl ether
47	NEG5931	247.1369	6.94	[M-H]-	247.1334	+14.21	79.9555 (100.0), 80.9631 (35.1), 248.0794 (18.6), 183.9990 (4.5), 68.7298 (2.6), 203.1422 (2.1), ...	0.003708	Terrecyclic Acid
48	POS20530	503.3056	4.61	[M+H]+	503.3068	-2.23	89.0602 (100.0), 133.0862 (56.2), 177.1123 (14.6), 87.0442 (8.3), 131.0708 (2.7), 90.0633 (2.4), ...	0.003665	3,6,9,12,15,18,21,24,27,30-Decaoxadotriacontane-1,32-diol
49	POS14208	339.1576	6.97	[M+Na]+	339.1572	+1.15	95.0860 (100.0), 81.0703 (64.1), 299.0217 (61.0), 109.1014 (60.9), 185.0267 (57.9), 107.0858 (56.4), ...	0.003662	2,4-dihydroxy 6-(5,7-dimethyl-2-oxo-trans-3-trans-5-nonadienyl)-3-methylbenzaldehyde
50	NEG10898	347.1525	6.62	[M-H]-	347.1495	+8.66	80.9633 (100.0), 79.9557 (43.9), 303.1945 (33.6), 311.2217 (33.4), 303.0435 (23.2), 301.0285 (22.7), ...	0.003646	6beta-methacryloyloxy-8,8-dihydroxy-1beta,10beta-epoxyeremophil-7(11)-ene-8,12-olide
51	POS8652	229.1178	1.45	[M+H]+	229.1188	-4.64	229.1557 (100.0), 70.0657 (89.0), 132.0654 (21.0), 142.0860 (10.6), 230.1569 (9.8), 196.0615 (6.6), ...	0.003620	Prolylhydroxyproline
52	NEG8798	307.0613	5.15	[M-H]-	307.0606	+2.15	239.0742 (100.0), 227.0331 (98.6), 229.1337 (30.6), 306.9899 (30.1), 307.1175 (26.0), 240.0805 (18.9), ...	0.003593	Dillanol
53	POS8536	227.1641	5.20	[M+H]+	227.1647	-2.77	109.1011 (100.0), 151.1123 (50.7), 209.1544 (50.5), 127.1111 (49.4), 123.0805 (39.4), 71.0499 (28.9), ...	0.003573	Annuionone G
54	NEG2998	185.0268	4.41	[M-H]-	185.0272	-2.32	79.9553 (100.0), 121.0637 (53.3), 138.8889 (24.0), 186.1119 (19.1), 141.0902 (12.2), 125.0959 (9.6), ...	0.003565	m-xylene-4-sulfonic acid
55	POS22596	576.3205	4.98	[M+H]+	576.3186	+3.31	103.0392 (100.0), 89.0603 (61.2), 133.0862 (38.2), 87.0442 (29.4), 177.1124 (11.2), 147.0643 (9.1), ...	0.003556	Ergokryptine
56	POS5162	173.1073	4.54	[M+H]+	173.1079	-3.31	144.0811 (100.0), 174.0760 (17.7), 146.0813 (13.5), 156.1020 (10.8), 57.0343 (9.1), 145.0838 (7.3), ...	0.003479	1,2,3,4-Tetrahydro-beta-carboline
57	POS12801	309.1871	8.06	[M+H]+	309.1913	-13.68	167.0880 (100.0), 150.1272 (39.3), 292.2289 (29.2), 111.0262 (27.7), 97.1020 (23.6), 125.0966 (21.8), ...	0.003478	(R)-1-O-b-D-glucopyranosyl-1,3-octanediol
58	NEG5970	248.0955	4.90	[M-H]-	248.0923	+13.05	124.0056 (100.0), 79.9553 (96.4), 106.9785 (61.1), 80.9632 (41.8), 161.0958	0.003371	Rigidiusculamide B

							(17.1), 136.9081 (16.9), ...		
59	POS13932	332.3301	8.05	[M+H]+	332.3317	-5.04	91.0543 (100.0), 240.2691 (44.6), 58.0658 (35.9), 241.2719 (5.1), 92.0579 (4.5), 59.0693 (0.6), ...	0.003369	Piptamine(antibiotic)
60	NEG9550	321.1338	7.13	[M-H]-	321.1338	-0.07	321.2089 (100.0), 277.1447 (97.9), 233.1534 (77.1), 321.1707 (52.9), 80.9633 (31.6), 165.0176 (30.5), ...	0.003180	[3aR-(3aalpha,6alpha,8abeta,9aalpha)]-5-[(Acetyloxy)methyl]-3a,4,6,7,8,8a,9,9a-octahydro-6-hydroperoxy-8a-methyl-3-methylenenaphtho[2,3-b]furan-2(3H)-one
61	POS15290	364.2835	5.82	[M+H]+	364.2852	-4.64	346.2748 (100.0), 255.2104 (93.8), 329.2443 (80.0), 365.1909 (75.1), 283.2415 (21.4), 311.2357 (18.6), ...	0.003173	(±)8(9)-EET Ethanolamide
62	NEG2932	183.0291	4.98	[M-H]-	183.0293	-1.52	83.0125 (100.0), 99.0068 (59.9), 139.0383 (44.1), 94.9150 (21.2), 138.9046 (16.7), 162.4914 (10.0), ...	0.003159	2-(3,4,5-trihydroxyphenyl)acetic Acid
63	POS10703	267.1698	3.75	[M+H]+	267.1709	-3.88	250.1444 (100.0), 72.0812 (60.3), 137.0594 (43.7), 179.0691 (16.5), 251.1456 (14.1), 120.0805 (8.2), ...	0.003156	Atenolol
64	NEG11463	359.1709	5.50	[M-H]-	359.1706	+0.94	341.1576 (100.0), 359.1144 (91.5), 115.0383 (76.6), 243.1214 (70.1), 279.1589 (58.5), 199.1329 (46.2), ...	0.003148	cropapine
65	POS12131	296.1273	6.83	[M+H]+	296.1287	-4.45	250.1214 (100.0), 251.1260 (14.3), 222.0925 (6.1), 232.1126 (2.5), 209.0823 (1.5), 278.2186 (1.1), ...	0.003140	Steporphine
66	NEG13879	421.2259	8.35	[M-H]-	421.2260	-0.19	80.9636 (100.0), 353.1974 (3.3), 227.1267 (0.7), 193.0848 (0.5), 67.9524 (0.5), 422.1113 (0.4), ...	0.003107	1,4-Bis(2-ethylhexyl) sulfosuccinate
67	NEG10217	334.1673	6.49	[M-H]-	334.1654	+5.63	100.0749 (100.0), 125.8714 (40.6), 306.1598 (31.0), 278.1532 (19.5), 298.1164 (14.5), 267.1065 (14.3), ...	0.003105	Doronenine
68	NEG3486	195.1018	5.33	[M-H]-	195.1021	-1.53	160.8400 (100.0), 195.8089 (99.9), 151.1113 (27.3), 95.0484 (10.2), 153.0285 (6.4), 93.0324 (3.5), ...	0.003104	Ficusic acid
69	NEG4092	209.0444	5.20	[M-H]-	209.0450	-3.01	121.0648 (100.0), 109.0275 (45.0), 165.0535 (33.3), 97.9990 (24.0), 93.0325 (23.3), 127.8691 (16.7), ...	0.003084	Clavatoic acid
70	POS9450	244.1537	4.62	[M+H]+	244.1549	-4.85	105.0703 (100.0), 86.0968 (74.0), 245.1330 (62.7), 145.1014 (48.8), 72.0816 (46.8), 226.1779 (43.5), ...	0.003014	N-3-Hydroxyoctanoyl-L-homoserine lactone
71	POS9583	246.1236	4.37	[M+H]+	246.1243	-2.65	228.1137 (100.0), 204.1125 (44.5), 83.0610 (27.0), 104.0502 (23.4), 229.1171 (12.9), 56.0504 (7.2), ...	0.002964	4-Acetamidoantipyrine
72	POS6987	201.0732	3.93	[M+H]+	201.0763	-15.36	202.1792 (100.0), 202.1054 (76.6), 93.0576 (62.5), 156.1020 (59.2), 101.0605 (53.5), 111.0440 (46.0), ...	0.002913	Astepyrone
73	POS16454	393.1915	7.47	[M+H]+	393.1922	-1.85	313.2344 (100.0), 311.2617 (56.6), 357.2671 (49.1), 375.2770 (23.9), 376.2568 (22.2), 339.2577 (16.7), ...	0.002882	(1R,3aS,3bS,5aS,7aS,11aR,11bS,11cS,13aR)-hexadecahydro-1-ethenyl-11b,13a-dimethyl-7H-cyclopenta[7,8]phenanthro[4,3-e][1,3,4]oxadithiin-7-one|krempene A
74	NEG4470	217.0418	5.26	[M-H]-	217.0420	-1.19	175.8730 (100.0), 62.9628 (57.4), 95.9149 (53.5), 217.1436 (45.7), 137.0587 (39.7), 127.8678 (36.0), ...	0.002879	2-Benzyl-4-chlorophenol
75	NEG3997	207.0113	4.77	[M-H]-	207.0116	-1.16	143.0489 (100.0), 103.9185 (63.5), 206.9711 (53.3), 87.9237 (51.9), 161.0435	0.002867	2-Naphthalenesulfonic acid

							(47.0), 127.8692 (45.4), ...		
76	NEG6273	255.0328	4.33	[M-H]-	255.0293	+13.50	211.0431 (100.0), 79.9554 (35.6), 255.0870 (16.7), 219.8436 (13.3), 212.0441 (9.2), 196.0197 (9.1), ...	0.002865	globosuxanthone D
77	POS9593	246.1696	4.20	[M+H]+	246.1705	-3.91	85.0289 (100.0), 86.0970 (53.7), 87.1004 (32.4), 187.0971 (29.7), 210.1483 (11.4), 60.0814 (10.9), ...	0.002827	Pivaloylcarnitine
78	POS17910	433.2545	8.63	[M+H]+	433.2590	-10.44	89.0603 (100.0), 133.0863 (55.2), 57.0708 (53.1), 71.0864 (49.5), 85.1018 (44.3), 307.1163 (29.3), ...	0.002796	Triflorestevione
79	POS9914	252.1948	7.91	[M+H]+	252.1964	-6.20	123.0679 (100.0), 197.1160 (83.0), 57.0708 (59.0), 150.0262 (45.2), 81.0703 (43.6), 141.0543 (41.4), ...	0.002741	Dihydroalprenolol
80	POS9045	236.1275	3.99	[M+H]+	236.1287	-5.12	190.1234 (100.0), 120.0813 (17.3), 119.0729 (13.0), 237.1575 (12.0), 166.0867 (11.1), 191.1259 (10.0), ...	0.002735	3',4'-Methylenedioxy-N-isopropylaminocathinone
81	POS11611	285.1846	7.12	[M+H]+	285.1855	-2.89	121.0647 (100.0), 151.1107 (21.0), 147.1167 (18.0), 267.1726 (12.4), 133.1008 (6.3), 122.0677 (6.1), ...	0.002717	Boldione
82	POS13644	327.0071	7.30	[M+H]+	327.0087	-4.81	98.9847 (100.0), 174.9923 (21.3), 250.9984 (4.1), 116.9947 (1.9), 89.0603 (1.7), 133.0862 (1.0), ...	0.002708	Tris(2-chloroisopropyl)phosphate
83	POS6628	195.1374	7.88	[M+H]+	195.1385	-5.56	195.0868 (100.0), 93.0703 (55.5), 89.0602 (48.8), 81.0702 (48.0), 149.1323 (38.7), 67.0549 (38.3), ...	0.002647	(S,Z)-Lyratol acetate
84	NEG10717	343.1916	7.36	[M-H]-	343.1909	+1.82	275.1684 (100.0), 205.0503 (76.0), 212.1204 (52.4), 325.1819 (47.9), 218.1287 (37.4), 189.1272 (34.1), ...	0.002629	3-Keto-16alpha-hydroxy-4-pregnen-18,20S-olid
85	NEG3399	193.0494	5.78	[M-H]-	193.0501	-3.75	121.0283 (100.0), 121.0637 (77.5), 148.0596 (55.3), 193.1014 (37.2), 149.0591 (27.0), 90.9319 (25.5), ...	0.002614	Monoethyl phthalate
86	POS17479	421.3152	8.91	[M+H]+	421.3165	-3.11	103.0392 (100.0), 103.0759 (58.6), 89.0603 (47.9), 57.0707 (41.2), 71.0863 (36.1), 59.0499 (34.3), ...	0.002600	SCHEMBL9230589
87	POS5276	175.0863	5.07	[M+H]+	175.0871	-4.61	134.0599 (100.0), 148.0963 (18.9), 104.0705 (7.4), 135.0635 (6.5), 158.0813 (5.2), 128.0707 (3.4), ...	0.002585	Indole-3-acetamide
88	NEG6599	261.0589	5.82	[M-H]-	261.0552	+14.41	246.0348 (100.0), 247.0376 (9.8), 79.9561 (8.7), 68.4420 (7.7), 146.9215 (6.8), 197.0977 (6.1), ...	0.002568	LMPK12130001
89	POS7573	211.0570	4.04	[M+H]+	211.0606	-17.19	211.1069 (100.0), 211.1449 (71.0), 165.1025 (37.2), 212.0913 (30.6), 193.0974 (24.9), 194.0794 (23.0), ...	0.002560	(3RS)-3,5,7-Trihydroxy-4,6-dimethyl-1(3H)-isobenzofuranon
90	NEG1730	153.0906	5.46	[M-H]-	153.0916	-6.30	93.0326 (100.0), 110.0354 (70.8), 95.0369 (47.2), 94.0364 (37.9), 153.0184 (36.8), 59.0122 (29.4), ...	0.002536	Boschnialactone
91	NEG12298	378.1014	6.24	[M-H]-	378.0978	+9.73	298.1458 (100.0), 299.1491 (8.8), 311.1326 (4.6), 379.2473 (2.5), 378.2336 (2.5), 378.1988 (1.9), ...	0.002487	12-Hydroxydihydrochelirubine
92	POS18244	442.3523	8.77	[M+H]+	442.3532	-2.08	443.2893 (100.0), 271.1552 (72.7), 89.0603 (58.5), 385.1764 (38.3), 121.0648 (37.4), 133.0862 (32.8), ...	0.002437	3-Hydroxy-11Z-octadecenoylcarnitine
93	NEG2759	179.1067	5.80	[M-H]-	179.1072	-2.99	179.9235 (100.0), 134.9868 (67.6), 90.9229 (26.3), 134.9126 (20.9), 178.9774 (19.2), 135.0806 (16.8), ...	0.002426	3-tert-Butyl-4-hydroxyanisole
94	POS15014	358.2002	5.58	[M+H]+	358.2018	-4.67	116.1070 (100.0), 72.0816 (37.0), 98.0968 (30.8), 256.2261 (14.5), 340.1895 (13.6), 91.0547 (6.3), ...	0.002396	(S)-Laudanosine
95	POS3835	152.1068	3.65	[M+H]+	152.1075	-4.99	153.0656 (100.0), 154.0491 (38.6), 152.0558 (26.7), 121.0647 (2.9), 153.0403 (2.8), 136.0394 (2.5), ...	0.002395	2-Acetyl-3-ethylidene-3,4,5,6-tetrahydropyridine
96	POS15028	358.2582	6.12	[M+H]+	358.2593	-3.09	137.1324 (100.0), 81.0701 (80.8), 95.0857 (78.6), 155.1428 (67.4), 60.0814 (26.4), 85.0288 (21.2), ...	0.002379	(7E)-5-Hydroxydodec-7-enoylcarnitine

97	NEG9046	311.1316	6.23	[M-H]-	311.1283	+10.37	311.1854 (100.0), 183.0108 (92.2), 267.1939 (39.4), 184.0188 (23.5), 293.1753 (17.0), 312.0019 (15.4), ...	0.002326	Nor-Dentatin
98	NEG9734	325.1690	7.36	[M-H]-	325.1651	+12.08	96.9583 (100.0), 265.1433 (22.0), 195.1740 (20.0), 307.1898 (17.7), 209.1170 (12.3), 122.9746 (7.6), ...	0.002314	6beta-acetoxy-7beta,8beta-dihydroxy-11alphaH-eremophilan-(12,8alpha)-olide
99	NEG11053	350.1635	5.81	[M-H]-	350.1617	+5.25	138.0220 (100.0), 196.0272 (10.9), 166.0167 (10.7), 106.9793 (9.7), 79.9557 (3.4), 124.0050 (3.2), ...	0.002284	Pirenzepine
100	POS18326	445.1183	9.34	[M+H]+	445.1206	-5.06	359.0275 (100.0), 91.0581 (71.9), 167.0552 (38.6), 445.3383 (36.3), 73.0478 (21.7), 360.0292 (16.7), ...	0.002279	Dodecamethylcyclohexasiloxane

Note: Putative annotations were obtained by matching the observed precursor m/z against the theoretical m/z of candidate molecular formulae in HMDB, MassBank, GNPS and an in-house spectral library, and were retained only when the accurate-mass match was combined with an MS/MS spectral match (<0.01 Da) (main text, Section 2.4; SI, Section 1.5). To make the mass accuracy of each individual annotation transparent, the assigned adduct ion, the observed and theoretical m/z and the resulting relative mass error (ppm) are reported for every compound. The 20 ppm window was used only as the initial database search window, and the actual mass errors of the retained annotations were much smaller (median |error| 4.1 ppm; 62% within ±5 ppm and 81% within ±10 ppm; maximum 19.5 ppm). “Theoretical m/z” is calculated from the molecular formula and the assigned adduct ion; “Mass error” is the relative deviation (ppm) between the observed and the theoretical m/z. “MS/MS fragment ions” lists up to the 6 most abundant product ions of the HCD MS/MS spectrum (m/z with relative intensity, %), with the surviving precursor ion and its ^{13}C isotopologue excluded; “...” indicates additional lower-abundance fragments and “n.d.” indicates that no MS/MS spectrum was recorded. All listed annotations correspond to putatively identified compounds (MSI Level 2); no authentic-standard-confirmed (MSI Level 1) assignments are claimed.

6 Cross-Validation Accuracy by Fold

6.1 Full Dataset (All Features, n=3,248)

The model trained on the complete feature set demonstrated exceptional performance. Across the five cross-validation folds, accuracy was perfect (100.0%) in four folds, with a single fold achieving 90.9% accuracy. The overall mean accuracy was 98.2% ± 3.6% (SD), corresponding to 55 correct predictions out of 56 total samples.

6.2 Without Pharmaceuticals (n=2,954)

When pharmaceutical compounds were excluded from the feature set, model performance remained very high but showed a slight, non-significant decrease. Accuracy was 100.0% in three folds, with two folds at 91.7% and 90.9%. The overall mean accuracy was 96.5% ± 4.3% (SD), with 54 correct predictions out of 56 samples.

6.3 Statistical Comparison

A Wilcoxon signed-rank test was performed to compare the accuracy distributions from the five folds between the two models (full dataset vs. dataset without pharmaceuticals). The resulting p-value of 1.000 indicates no statistically significant difference in the predictive performance of the two models.

6.4 Confusion Matrix (Full Dataset, Consensus)

The consensus confusion matrix for the model trained on all features reveals near-perfect classification. Six of the seven catchments (HB, LB, LC, LS, SY, TD) were predicted with 100% accuracy (8/8 correct). A single misclassification occurred: one sample from the MD catchment was incorrectly predicted as belonging to the TD catchment. This resulted in an overall accuracy of 98.2% (55/56 correct).

6.5 Sensitivity analysis

As a sensitivity check, all core analyses were repeated using Euclidean distance on the identical standardized data, yielding qualitatively consistent results (distance ratio 1.29 vs. 1.65 for Bray–Curtis; $p < 0.001$ in both cases), indicating that the spatial structure is robust to the choice of distance metric.

Additional Notes

Data Availability: All raw data, processed data, and analysis scripts are available upon request from the corresponding author.

Code Availability: Analysis code is available upon request from the corresponding author.

Python version: 3.9.12

Scikit-learn: 1.1.1

XCMS: 3.18.0

scipy: 1.9.0

pingouin: 0.5.2

MSDIAL: 4.90
